# Aerobic exercise-induced changes in fluid biomarkers in Parkinson’s disease

**DOI:** 10.1038/s41531-025-01042-8

**Published:** 2025-07-01

**Authors:** Nijee S. Luthra, Niyati Mehta, Miranda J. Munoz, Giamila Fantuzzi, Guillaume Lamotte, Jacob M. Haus, Nikolaus R. McFarland, Malú G. Tansey, Paulina Gonzalez-Latapi, Gabriela Caraveo, Un Jung Kang, Daniel M. Corcos

**Affiliations:** 1 Department of Neurology, University of California San Francisco, San Francisco, CA USA; 2 Department of Physical Therapy and Human Movement Sciences, Northwestern University, Chicago, IL USA; 3 Department of Kinesiology and Nutrition, University of Illinois at Chicago, Chicago, IL USA; 4 Department of Neurology, University of Utah, Salt Lake City, UT USA; 5 School of Kinesiology, University of Michigan, Ann Arbor, MI USA; 6 Department of Neurology, University of Florida, Gainesville, FL USA; 7 Fixel Institute for Neurological Diseases, University of Florida Health, Gainesville, FL USA; 8 Stark Neuroscience Research Institute, Indiana University School of Medicine, Indianapolis, IN USA; 9 Department of Neurology, Northwestern University, Chicago, IL USA; 10 Department of Pharmacology, Northwestern University, Chicago, IL USA; 11 Departments of Neurology and Neuroscience, NYU Grossman School of Medicine, New York, NY USA

**Keywords:** Parkinson's disease, Parkinson's disease

## Abstract

Parkinson’s disease (PD) is a neurodegenerative disease characterized by motor and non-motor symptoms that progressively deteriorate and for which there is no disease-modifying pharmacological treatment. Exercise is widely recommended for individuals with PD due to its potential neuroprotective benefits. However, the mechanisms underlying these exercise-induced effects in PD remain poorly understood. Analyzing fluid biomarkers responsive to exercise could offer valuable insights into the mechanisms by which exercise impacts PD and aid in optimizing exercise prescriptions for individuals with PD. This review explores exercise-responsive biomarkers categorized into three key groups—neurotrophic, inflammatory, and neuroendocrine markers. It highlights both well-validated biomarkers and candidates with promising potential. We also highlight key biomarkers linked to PD pathology, such as α-synuclein, and their potential connection to exercise based on current evidence. Comprehensive characterization of these biomarkers will advance our understanding of the biological effects of exercise in PD, enabling mechanism-based and objective measures to evaluate exercise response in future clinical trials and its impact on PD signs and symptoms.

## Introduction

The prevalence of PD has doubled over the past 25 years and is projected to exceed 12 million cases by 2040^1,2^. This rapid increase is driven by an aging population with longer lifespans and environmental factors such as pollution. PD is an extremely complex disorder with numerous etiological factors, diverse pathogenic pathways, and a myriad of motor and non-motor symptom manifestations that vary in progression between individuals^3^. Strategies targeting specific pathogenic pathways have thus far been unsuccessful in modifying disease progression^4,5^. In this review, we explore a therapy with broad therapeutic potential – aerobic exercise – and discuss how it may influence multiple pathogenic pathways simultaneously. Specifically, we discuss the changes in circulating biomarkers in the context of PD and in response to exercise to provide insights into how exercise can impact brain health and PD pathogenesis.

## Complexity of PD

One of the major challenges in PD is its heterogeneity, with signs and symptoms encompassing motor, cognitive, behavioral, autonomic, and sleep disturbances, as well as variability in the rate of disease progression^6^. Although the majority of cases of PD appear to be sporadic, evidence supports predisposing factors including environmental toxic exposure and genetic defects^7^. The hallmark motor symptoms primarily stem from the progressive loss of nigrostriatal dopaminergic neurons. However, other brain regions are significantly affected and are responsible for treatment-resistant motor and non-motor symptoms, which often manifest decades before the onset of motor signs^8^. Neurodegeneration in PD may be a result of diverse pathogenic pathways, including the toxicity of soluble and insoluble aggregated α-synuclein, mitochondrial dysfunction, defective proteolysis due to ubiquitin-proteasome system dysfunction, oxidative stress, and inflammation^9–11^.

Current strategies to modify disease progression in PD have included agents targeting α-synuclein accumulation or cell-to-cell transmission, specific organelles such as mitochondria and lysosomes, associated proteins like β-glucocerebrosidase and leucine-rich repeat kinase 2, neuronal rescue pathways such as calcium channel blockers and iron reducers, and molecules involved in neuroinflammatory pathways^4^. Despite these efforts, no pharmacological treatment has proven effective in modifying disease progression.

## Exercise and Clinical Symptoms of PD

Multiple clinical studies and meta-analyses highlight the benefits of various exercise modalities on improving motor symptoms, muscle strength, functional mobility and balance, and quality of life in individuals with PD^12,13^. Most studies have focused on three main interventions: aerobic exercise, resistance exercise, and balance exercises. Additional studies have investigated the effects of mind-body practices such as Tai Chi, Yoga, and Health Qigong^14,15^. Parameters such as the intensity (low, moderate, or high), frequency, muscle contraction types (e.g., eccentric vs. concentric), interval vs. continuous training, and the setting (home vs. supervised) vary by exercise modality and study design. It is beyond the scope of this review to cover all exercise types, so we direct readers to published reviews on other exercise modalities and their effects on PD symptoms^13,16–18^. Furthermore, practical guidelines for exercise prescriptions are available^19^.

The present review focuses on long-term (chronic) aerobic exercise training, as it is the form of exercise that has the most compelling evidence for slowing disease progression and reducing symptoms in PD, based on extensive preclinical studies and clinical studies^20–23^. The primary goal of aerobic exercise training is to enhance the capacity of the circulatory and respiratory systems to supply oxygen to skeletal muscles and to sustain prolonged, rhythmic activity. Activities such as cycling, running, rowing, brisk walking, and swimming achieve this by increasing breathing and heart rates.

Longitudinal observational cohort studies have demonstrated that individuals with PD who exercise regularly experience a slower progression of motor and cognitive symptoms and report better quality of life outcomes compared to non-exercisers^24–26^. Furthermore, strong evidence suggests that exercise reduces the risk of developing clinical PD^27,28^, potentially by influencing fundamental pathogenic processes. To date, three randomized controlled trials have shown that moderate (50-70% of maximum heart rate or heart rate reserve) to high-intensity (80–85% of maximum heart rate or heart rate reserve) aerobic exercise has the potential to slow disease progression and reduce the signs and symptoms of PD^20–22^. Data from these trials also suggest a dose-response relationship between aerobic exercise intensity and motor function improvements in PD, with higher aerobic exercise dosage (number of sessions x session duration) yielding greater effects on motor symptoms. However, evidence is needed to confirm this relationship.

A recent study by De Laat et al. demonstrated that 6 months of high-intensity exercise in patients with mild and early PD (*n* = 10) reversed the expected decrease in dopamine transporter availability on ^18^F-FE-PE2I PET imaging, showing a significant increase in both the substantia nigra and putamen^29^. Additionally, exercise reversed the expected decline in neuromelanin concentration in the substantia nigra, resulting in a significant increase. These findings suggest that exercise may enhance the dopaminergic system and deserves further investigation.

Studies examining the effect of aerobic exercise on cognition in PD have yielded conflicting results. While some studies reported improvement in cognitive function^30,31^, others showed no significant changes^21,32^. Similarly, there is insufficient evidence to support a beneficial effect of aerobic exercise training on non-motor symptoms of PD, including depression, apathy, sleep disturbances, fatigue, or constipation^33^. Exercise, however, has the potential to improve dysregulated crosstalk between the brain and peripheral systems and to improve both motor and non-motor symptoms, many of which are thought to originate in the periphery^34–36^. Assessing biomarker changes in conjunction with symptom improvement will provide stronger mechanistic evidence for the role of exercise in PD.

## A Role for Biomarkers in Understanding the Effects of Exercise

Given the complexity surrounding biomarker definitions and their application in research and clinical practice, the NIH and FDA have created the “Biomarkers, Endpoints, and other Tools” (BEST) resource. The BEST resource categorizes biomarkers into seven types along the clinical continuum of disease: risk/susceptibility, diagnostic, predictive, prognostic, monitoring, safety, and response^37^. Among these, response biomarkers indicate a biological response to a therapy or environmental agent. This review focuses on response biomarkers that may reflect the diverse mechanisms by which exercise benefits individuals with PD. Some biomarkers may have application in different categories. There have been promising developments in detecting abnormal α-synuclein for diagnosis of PD with Lewy bodies as described in a later section. However, the reliability of other biomarker categories, such as risk/susceptibility, predictive/prognostic and monitoring biomarkers, remains under investigation in PD and will not be discussed here.

**Fig. 1:**
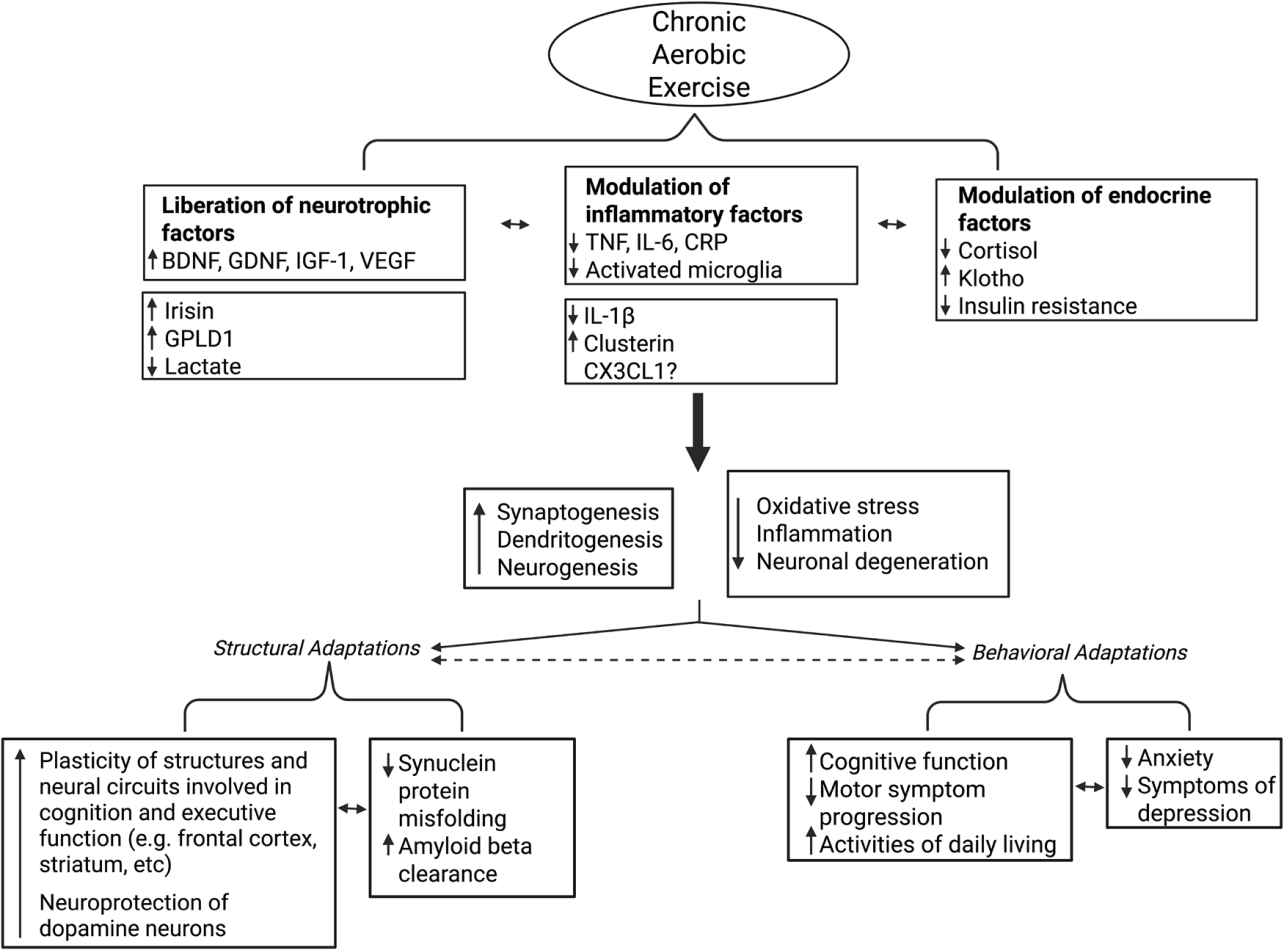
Proposed Model for Exercise-Induced Changes in Biomarkers and their Mechanisms in PD. Chronic aerobic exercise leads to liberation of neurotrophic factors, modulation of inflammatory factors, and modulation of neuroendocrine factors. Downstream mechanisms may include enhanced neuronal synthesis, structural and behavior adaptations. Adapted from Paillard et al. 2023. BDNF brain-derived neurotrophic factor, GDNF glial cell line-derived neurotrophic factor, IGF-1 insulin-like growth factor 1, VEGF vascular endothelial growth factor, GPLD1, glycosylphosphatidylinositol-specific phospholipase D1, TNF tumor necrosis factor, IL-6 interleukin-6, CRP C-reactive protein.

Exercise has demonstrated neuroprotective effects in animal models of PD^23,38–40^. These effects are thought to result from neurogenesis, synaptogenesis, and angiogenesis and from modulation of mitochondrial function, oxidative stress, and neuroinflammation^41–43^. Identifying exercise-responsive biomarkers that influence these potentially disease-modifying mechanisms is crucial. The ongoing SPARX3 (Study in Parkinson’s Disease of Exercise Phase 3 Clinical Trial) aims to identify key biomarkers that respond to moderate- and high-intensity aerobic exercise in people with PD^44^. Figure 1 presents an overview of mechanisms involved in the beneficial effects of chronic aerobic exercise.

## Exercise-Induced Changes in Neurotrophic/Neuroprotective Markers

While the pathophysiological mechanisms underlying PD remain unclear, it is well-established that neurological symptoms are a consequence of dopaminergic neuronal loss^45^. Extensive research has focused on the role of neurotrophic factors as protective agents against neuronal damage in PD^46,47^. Neurotrophic factors are secreted peptides that play key roles in development, proliferation, protection, survival, and restoration of neuronal cells. These factors have shown potential for disease modification in preclinical PD models, and clinical studies have linked changes in neurotrophic factors levels to motor and non-motor symptoms of PD^46–50^.

In individuals with PD, alterations in neurotrophic factors have been reported in post-mortem brains, cerebral spinal fluid (CSF), and blood. Specifically, brain-derived neurotrophic factor (BDNF), nerve growth factor (NGF), vascular endothelial growth factor (VEGF), and glial cell line-derived neurotrophic factor (GDNF) levels have been reported to be decreased in post-mortem PD brains^51–55^ and CSF^56^, as well as in peripheral blood^57–60^. Conversely, cerebral dopamine neurotrophic factor (CDNF) and insulin-like growth factor 1 (IGF-1) levels are elevated in post-mortem brains and CSF, likely as part of a compensatory protective mechanism^54,61^. Similarly, serum levels of IGF-1 and mesencephalic astrocyte-derived neurotrophic factor (MANF) are increased in PD^61,62^. Together, this evidence suggests that both peripheral and central neurotrophic factor levels are dysregulated in PD.

**Fig. 2:**
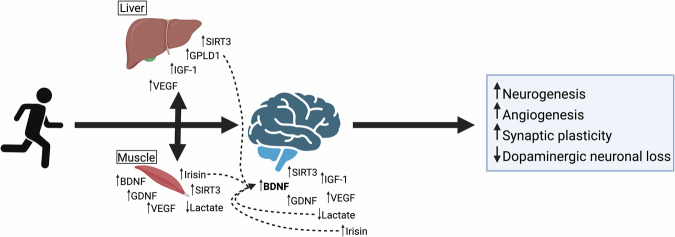
Exercise-Induced Changes in Neurotrophic/Neuroprotective Factors. Chronic aerobic exercise stimulates elevations in multiple neurotrophins, with a most robust response in BDNF, VEGF, and IGF-1 in the periphery and in the CNS. Irisin, GPLD1, SIRT3, and lactate are involved in tissue-brain crosstalk to mediate positive benefits of exercise, with one mechanism being an increase in neurotrophins such as BDNF by GPLD1. Elevation in neurotrophic factors has been linked to pathways that counter neurodegeneration and synaptic plasticity. BDNF brain-derived neurotrophic factor, GDNF glial cell line-derived neurotrophic factor, IGF-1 insulin-like growth factor 1, VEGF vascular endothelial growth factor, GPLD1 glycosylphosphatidylinositol-specific phospholipase D, SIRT3 sirtuin-3. Created in BioRender. Mehta, N. (2025) https://BioRender.com/a05k823.

Neurotrophic factors represent a leading category of biomarkers that mediate many of the benefits of exercise. In animal models, exercise robustly increases levels of BDNF, GDNF, VEGF, IGF-1, NGF, and CDNF^41,50,63,64^. The effects depend on exercise type, intensity, and whether changes are acute or chronic. In this review, we focus on BDNF, GDNF, VEGF and IGF-1 as principal neurotrophic factors to assess responses to chronic exercise because their levels are altered in PD^51–62^. Their circulatory levels consistently increase with long-term exercise, and these growth factors exert complementary effects, supporting neuronal survival and regulating brain plasticity and function (Fig. 2). Additionally, we discuss other biomarkers, including irisin, glycosylphosphatidylinositol-specific phospholipase D1 (GPLD1), sirtuin-3 (SIRT3), and lactate. Emerging evidence suggests these biomarkers respond to chronic exercise, potentially mediating peripheral tissue-brain crosstalk, and they have neuroprotective effects.

### BDNF

BDNF plays a key role in neuronal maturation and survival, adult hippocampal neurogenesis, and neural plasticity^65^. Experimentally, inhibition of BDNF mRNA expression via antisense oligonucleotide infusions results in the loss of nigral dopaminergic neurons^66^, providing evidence that reduced BDNF may contribute significantly to the degeneration of dopaminergic neurons in PD. Conversely, boosting BDNF expression through gene therapy in PD rat models has been demonstrated to prevent the loss of nerve terminals and cell bodies of the nigrostriatal dopaminergic pathway, while also inducing sprouting of dopaminergic fibers^67,68^. In clinically and neuropathologically typical PD, BDNF mRNA expression is reduced by 70% in the substantia nigra pars compacta (SNpc), attributed to both the loss of dopaminergic neurons that produce BDNF and reduced BDNF mRNA expression in surviving dopaminergic neurons^69^. Decreased levels in BDNF have also been reported in the blood of individuals with PD^57,60^. Moreover, BDNF levels have been found to negatively correlate with age at PD onset and with non-motor symptoms such as impaired cognition and depression^70,71^. Interestingly, serum BDNF levels positively correlate with a longer duration of disease, increased symptom severity, and more advanced stage of PD^72^. One possible explanation is that lower BDNF levels may contribute to the initial risk and pathogenesis of PD risk, while increased levels during disease progression could represent a compensatory mechanism. This dual role - both a reduction and a compensatory increase in BDNF - suggests that the effects of exercise on BDNF may depend on the stage of the disease.

It is well established that exercise increases BDNF in skeletal muscle, promoting paracrine effects that influence motor neuron innervation^73^. Voluntary exercise in mice increases BDNF levels in the dorsal striatum and stimulates dopamine release^74^. BDNF is also necessary for exercise-induced increases in hippocampal dentate gyrus, which enhances memory and learning^75,76^. In animal models of PD, alleviation of motor deficits following exercise is directly linked to increased BDNF levels^39,77^. There is robust evidence of an increase in blood BDNF levels with chronic aerobic exercise in healthy adults^78–80^. A recent systematic review by Paterno et al. analyzed 16 studies (8 two-arm trials and 8 single-arm trials) involving 370 PD participants and found significant rises in serum or plasma BDNF levels after 4–12 weeks of chronic aerobic or multimodal exercise, including aerobic training^81^. Both exercise intensity and total exercise volume were positively associated with BDNF changes. Similarly, Kaagman et al. conducted a meta-analysis of 5 trials (*n* = 216 participants with PD), revealing significant changes in serum or plasma BDNF levels with chronic aerobic training^82^. Furthermore, BDNF changes correlated with improvements in motor performance, measured by the Unified Parkinson’s Disease Rating Scale (UPDRS) part III motor score, as well as ambulatory capacity and balance^82^. These findings suggest that BDNF may play a key role in mediating the motor benefits of exercise in PD.

### GDNF

GDNF is expressed in the striatum and other regions that receive dopaminergic input from SNpc and motor neurons. Two studies have reported lower serum GDNF levels in individuals with PD compared to healthy controls, with this reduction associated with cognitive impairment^59,83^. Additionally, post-mortem analyses have shown reduced GDNF levels in the hippocampi of PD brains, despite no evidence of neuronal loss in that region^54^. Given its neuroprotective potential, GDNF administration has been explored as a therapeutic strategy in preclinical models of PD, where it has been shown to prevent nigral neuron loss and restore disease-associated neurochemical and behavioral changes^84,85^. Similarly, in Rhesus monkeys with toxin-induced parkinsonism, GDNF administration significantly improved postural instability, rigidity, and bradykinesia, while also enhancing dopamine levels and increasing dopaminergic fiber density^86^. Clinical trials investigating intraputaminal GDNF delivery in PD have reported marked improvements in UPDRS motor scores and “OFF” times in open-label studies^87,88^. However, subsequent double-blind, placebo-controlled trials did not show significant UPDRS motor score improvements and were discontinued due to excessive weight loss^89–91^. These findings suggest that while GDNF administration may offer benefits in PD, it is likely insufficient as a standalone treatment. Exercise, in contrast, may provide broader therapeutic effects, potentially including GDNF elevation as one of its mechanisms.

Chronic aerobic exercise has been shown to increase GDNF levels in the striatum, hippocampus, spinal cord, and muscle tissue in rodent models^92–96^. However, human studies on the effects of exercise on circulating GDNF levels remain limited. To date, only one prospective cohort study has demonstrated higher serum GDNF levels in healthy adults who engaged in aerobic exercise for six months compared to their sedentary counterparts^97^. Further research is needed to determine how circulating GDNF levels respond to aerobic exercise in individuals with PD.

### IGF-1

Alongside BDNF and GDNF, IGF-1 plays a crucial role in mediating exercise-induced benefits on neuroplasticity. IGF-1 signaling regulates pathways that promote cell growth and survival, maturation, and proliferation, supporting tissue maintenance and repair^98^. Additionally, IGF-1 promotes angiogenesis and amyloid clearance^99,100^. IGF-1 has also been shown to enhance exercise-induced BDNF signaling, contributing to improvements in learning and memory^101^. Notably, blocking peripheral IGF-1 entry into the brain abolishes the neurogenic effects of exercise^102^.

In PD, multiple studies show that serum IGF-1 levels are elevated at diagnosis, possibly as a compensatory mechanism, but this increase diminishes as the disease progresses^103–106^. One study also reported elevated CSF IGF-1 levels at baseline in PD^61^. Lower serum IGF-1 levels at disease onset are associated with worse prognosis, increased cognitive impairment risk, and faster disease progression, suggesting that individuals who fail to exhibit an adequate IGF-1 increase experience poorer outcomes^105,107^. Several studies have examined the effects of chronic aerobic exercise on IGF-1 levels. Baker et al. found that a 24-week aerobic exercise program increased plasma IGF-1 levels in men with mild cognitive impairment (MCI), but not in women, and was associated with cognitive improvements^108^. In a meta-analysis, Nasir et al. reported a significant increase in IGF-1 in postmenopausal women following long-term aerobic exercise^109^. Other studies have reported either maintenance of IGF-1 levels following 7-24 weeks of aerobic exercise^110–112^ or a decrease after 48 weeks of moderate-intensity aerobic exercise^113^. Discrepancies in findings may stem from differences in comorbidities, intervention duration, and exercise intensity. Studies evaluating IGF-1 changes in response to exercise in PD remain limited. To date, only Stuckenschneider et al. have reported a slight improvement in serum IGF-1 levels following an 8-week multimodal exercise intervention (aerobic + resistance + balance/dual task) in individuals with PD^114^.

### VEGF

VEGF plays a central role in brain angiogenesis, promoting neuronal survival and glial growth^115^. Like IGF-1, VEGF is essential for exercise-induced neurogenesis, as its peripheral blockade abolishes running-induced neurogenesis^116^. VEGF also exerts potent neuroprotective effects on dopaminergic neurons, likely through its angiogenic and glial-proliferative properties^117^. Notably, low-dose administration of VEGF-secreting cells provides significant neuroprotection when implanted into the striatum of PD rodent models^118^. Serum VEGF levels in individuals with PD have not shown to differ significantly from controls^119^. However, CSF levels of VEGF have been shown to be elevated in people with PD and PD dementia compared to healthy controls^56^. This increase is associated with greater gait difficulty, orthostatic hypotension, and increased blood-brain barrier (BBB) permeability. Higher CSF VEGF levels align with findings in postmortem PD brains, which exhibit increased angiogenesis^120,121^, and recent studies suggest that disruptions in these pathways contribute to disease progression^56,122^.

Experimental evidence indicates that exercise induces VEGF upregulation^123,124^. In rodent models, exercise enhances vascularization and VEGF levels in the substantia nigra, whereas aging and sedentary behavior are linked to reduced nigral microvascular density and VEGF mRNA expression^125^. A systematic review and meta-analysis by Song et al., which included 28 studies on chronic aerobic exercise in older adults, found that blood VEGF concentrations are higher post-exercise^126^. While no significant effect of exercise duration was observed, a subset of studies (*n* = 21) reported a trend toward increased VEGF levels following ≥4 weeks of aerobic exercise compared to a single exercise session^126^. These studies suggest that chronic exercise can enhance VEGF levels, potentially contributing to its neuroprotective effects in PD. However, more studies are needed to determine how VEGF changes with exercise in people with PD.

### Irisin

Irisin is a myokine primarily expressed in skeletal muscle during exercise^127^. A secretory form of the transmembrane protein fibronectin type III domain containing 5, irisin has gained significant attention since its discovery in 2012 because of its role in regulating energy metabolism and its links to metabolic and neurodegenerative disorders^128,129^. It can cross the BBB and directly induce expression of hippocampal BDNF and other neuroprotective genes^130,131^. Additionally, irisin has been shown to protect against neuronal injury in ischemic conditions^132,133^. In preclinical models of PD, irisin administration rescues dopaminergic neurons from degeneration, restores mitochondrial biogenesis, and alleviates oxidative stress^134,135^. Kam et al. have shown that intravenous irisin delivery via viral vectors following intrastriatal injection of α-synuclein preformed fibrils in mouse models of PD reduces pathological α-synuclein accumulation, preserves dopamine neurons, prevents striatal dopamine depletion, and improves motor deficits^136^. In people with PD, Shi et al. reported that plasma irisin levels decline as the disease progresses, negatively correlating with plasma α-synuclein levels and positively correlating with dopamine uptake in the striatum^137^.

An increasing number of clinical trials are investigating how irisin responds to exercise in humans. Jandova et al. conducted a systematic review and meta-analysis on 51 studies examining chronic exercise in both healthy adults and individuals with conditions such as interstitial lung disease, progressive multiple sclerosis (MS), or type 2 diabetes^138^. Among these, 25 were randomized controlled trials. Within-group analyses showed that blood irisin levels increased in 23 studies and decreased in 10, with a statistically significant between-groups effect in 15 studies. Chronic aerobic training alone demonstrated a significant positive effect in 4 studies compared to non-exercise control groups^138^. The considerable heterogeneity in findings may stem from differences in training protocols, participant populations, and methodological aspects of irisin measurement^138^. One major challenge in clinical trials is the accuracy of circulating irisin measurements. The commonly used ELISA kit has been criticized for its low reliability due to variability in available antibodies. Albrecht et al.^139^ highlighted this issue, cautioning that ELISA-based results should be interpreted carefully until the reliability of irisin antibodies is validated. While mass spectrometry is more accurate, it requires complex, multi-step sample preparation that introduces additional variables.

Only one study thus far has evaluated the effects of chronic exercise on irisin levels in people with PD. Zhang et al. have shown that 12 weeks of regular aerobic exercise increased serum irisin levels, which positively correlates with improved balance function^134^.

### GPLD1

GPLD1 is another exercise-induced circulating factor that may be crucial in the crosstalk between peripheral tissues and the brain during exercise^140^. As a hepatokine, GPLD1 has been implicated in neurogenesis and several neurodegenerative diseases^141^. In a novel approach, Horowitz at el. demonstrated that the transferring of plasma from exercised aged mice to sedentary aged mice conferred exercise-related benefits on adult neurogenesis and increased BDNF levels^142^. The study identified GPLD1 as a blood factor enriched in the transferred plasma and linked it to improved cognitive performance. Moreover, in vivo liver transfection to elevate GPLD1 levels led to increased BDNF expression in the hippocampus of aged mice^142^. Interestingly, GPLD1 does not readily cross the BBB, suggesting it mediates brain benefits indirectly through peripheral-brain signaling^140^. In humans, GPLD1 levels are higher in physically active elderly individuals (average daily steps ≥7100) compared to their sedentary counterparts (average daily steps <7100)^142^. However, no randomized controlled trials have yet examined the effects of exercise on GPLD1 in healthy adults or individuals with PD.

### SIRT3

SIRT3 is gaining recognition as a potential neuroprotective factor in neurodegenerative diseases such as PD. As a key regulator of mitochondrial homeostasis, SIRT3 has been identified as a potential disease-modifying target, given that mitochondrial dysfunction is a hallmark of PD pathology^143^. By deacetylating mitochondrial enzymes, SIRT3 prevents age-related mitochondrial dysfunction and oxidative stress^144^. Additionally, it supports mitophagy to clear damaged mitochondria, induces autophagy in macrophages, and regulates NLRP3 inflammasome activation, contributing to neuroprotection and anti-inflammatory effects^145,146^.

Numerous studies have linked SIRT3 to longevity in humans and rodents^147–149^. Trinh et al. have reported that SIRT3 levels are reduced by over 50% in the SNpc of PD participants compared to healthy controls^150^. In rodent models of PD, SIRT3 overexpression prevents dopaminergic cell loss, decreases reactive oxygen species (ROS) production, and mitigates oxidative stress^151–153^. Oligomeric α-synuclein associates with mitochondria and reduces SIRT3 levels, whereas SIRT3 overexpression has been shown to reduce α-synuclein oligomer formation^154^.

Given its role in cellular aging, SIRT3 is increasingly studied in the context of exercise, a well-known anti-aging intervention^155^. Chronic aerobic exercise (≥8 weeks) increases SIRT3 in skeletal muscle and serum across all age groups^156,157^. Notably, SIRT3 expression is nearly 40% lower in sedentary older adults but remains elevated in aerobic-trained individuals, regardless of age^158^. Investigating SIRT3 response to aerobic exercise may provide further insight into its role as a key mediator of exercise-induced benefits in PD.

### Lactate

Evidence over recent years has also pointed to lactate as an important mediator for the benefits of exercise on brain health^159^. Lactate was once considered a waste product responsible for muscle fatigue, but it is now recognized as a metabolic signaling molecule critical for brain function. It plays a crucial role in energy delivery, storage, production, and utilization^160,161^. Lactate exhibits dual roles: on one hand, it can be detrimental by increasing ROS production and oxidative stress; on the other hand, it is vital for maintaining brain homeostasis, promoting synaptic plasticity, neurogenesis, angiogenesis, and anti-inflammatory effects^162^. Liguori et al. have reported elevated CSF lactate levels in individuals with early PD, which correlated with clinical disease progression and neurodegeneration markers such as phosphorylated-tau^163^. Conversely, other studies have found no significant changes in serum lactate levels in individuals with moderately advanced PD^164^.

Chronic exercise induces metabolic adaptations, particularly in skeletal muscle, leading to reduced lactate production at a given intensity^165^. In people with PD, Martino et al. demonstrated that after 4 weeks of treadmill training, acute elevation in lactic acidemia is less pronounced^166^. Similar normalizations in lactate levels has been observed with chronic exercise in people with MS^167^ and type 2 diabetes^168^. However, further research is needed to confirm the specific effects of chronic exercise on lactate regulation in PD and its correlation with clinical symptoms.

## Exercise-Induced Changes in Inflammatory Markers

Substantial evidence supports a pathogenic role of inflammation in PD. However, it remains unclear whether the initial source of inflammation originates in the central nervous system (CNS) or the periphery, as immune alterations are present in both compartments, even in early PD^169^. Notably, chronic inflammatory conditions such as inflammatory bowel disease (IBD), psoriasis, arthritis, and diabetes—each linked to increased PD risk—suggest that peripheral inflammation may act as a trigger for neuroinflammation in PD^170^.

In individuals with PD, certain inflammatory biomarkers are significantly altered in both the periphery and the CNS, while others show changes in only one compartment. A systematic review and meta-analysis of 152 studies reported elevated levels of cytokines IL (interleukin)-1β, IL-6, tumor necrosis factor (TNF), the chemokine CCL2 (MCP1), and C-reactive protein (CRP) in both peripheral blood and CSF of individuals with PD compared to healthy controls^171^. Conversely, CX3CL1 (fractalkine), CXCL12, soluble TNF receptor-1 (sTNFR1), and N-terminal-pro-B-type natriuretic peptide (NT-proBNP) were significantly increased only in peripheral blood, whereas nitric oxide was significantly elevated only in CSF^171^. Interferon (IFN)$\alpha$2, IFNγ, and IL-4 were significantly decreased in peripheral blood^171^. While these findings may reflect true differences between peripheral and central inflammation in PD, they could also be influenced by statistical power limitations (fewer studies on certain mediators) or technical variables such as assay sensitivity and timing of blood collection.

Although strenuous acute physical exercise can be pro-inflammatory^172^, chronic exercise consistently reduces inflammation across various populations, including individuals with type 2 diabetes, metabolic disorders, and aging-related conditions^173–176^. In these populations, exercise significantly lowers peripheral blood levels of TNF, IL-6, and CRP—biomarkers also elevated in PD. Small studies in individuals with PD have shown that moderate- to high-intensity interval training (8–12 weeks) significantly reduces TNF levels in peripheral blood, though no effect has been observed for IL-6, and CRP remains unexamined^177,178^. It is also unclear whether exercise-induced reductions in peripheral inflammation extend to the CNS. However, extensive preclinical evidence indicates that exercise reduces neuroinflammation, particularly IL-1β and TNF, in brain regions implicated in PD^179^, suggesting that similar mechanisms may apply to humans^177–179^.

**Fig. 3:**
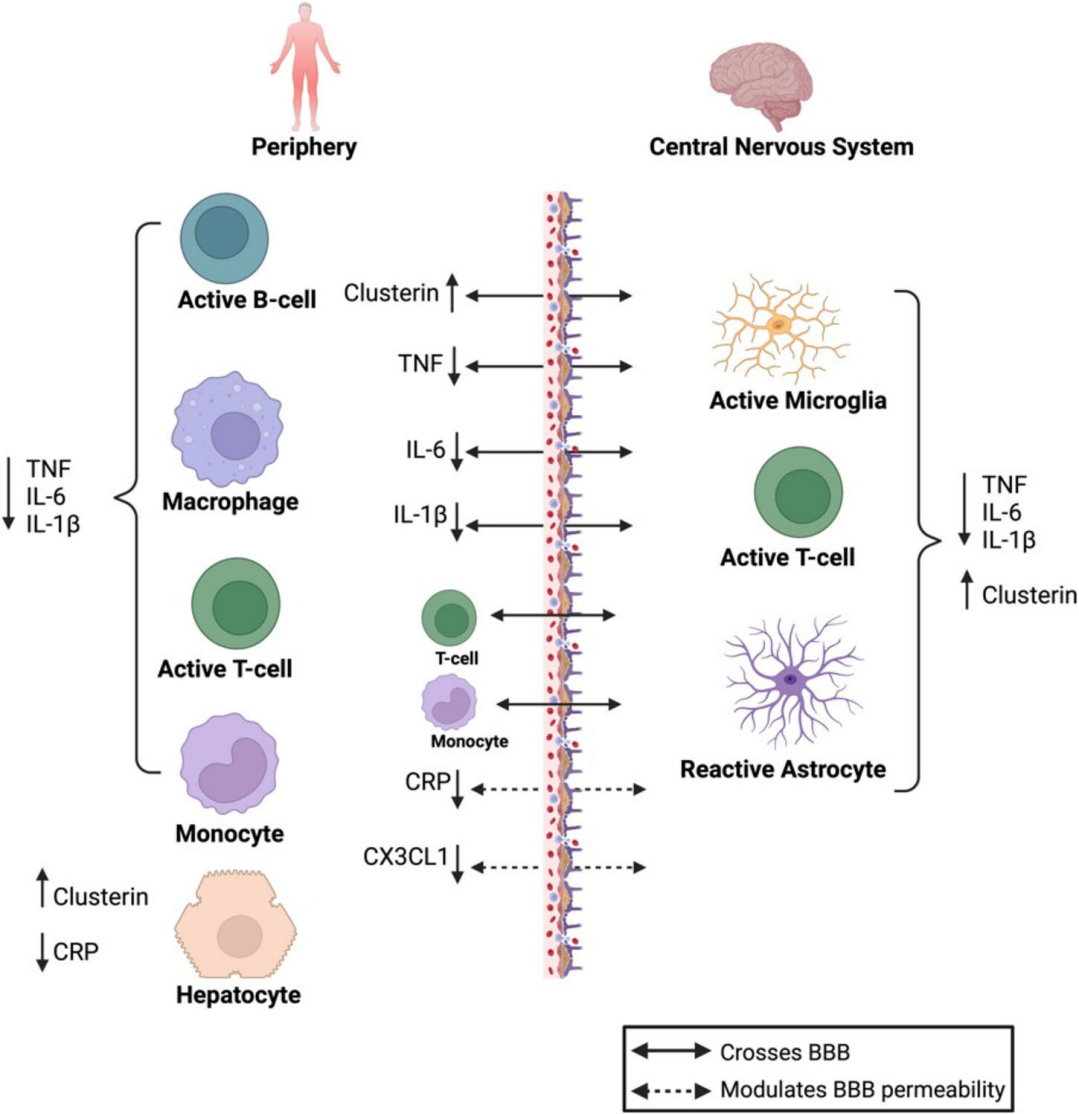
Expected exercise-induced changes in inflammatory markers. There are chronic increases in inflammatory markers in both central nervous system and periphery in PD. There is increased permeability of the BBB in PD, and many inflammatory markers including IL-6, TNF, IL-1β, and clusterin can cross the BBB, while others such as CRP and CX3CL1 modulate the permeability of the BBB in PD. Chronic aerobic exercise may be effective at reducing levels of inflammatory markers in both the central nervous system and periphery. PD Parkinson’s disease, BBB blood brain barrier, IL-6 interleukin-6, TNF tumor necrosis factor, IL-1β interleukin-1 beta, CRP c-reactive protein, CX3CL1, fractalkine. Created in BioRender. Mehta, N. (2025) https://BioRender.com/d17z951.

Three primary reasons support the use of TNF, IL-6 and CRP as robust biomarkers for assessing the effects of exercise on inflammation in PD: 1) each of these three markers is significantly elevated in both peripheral blood and CSF in individuals with PD versus controls^171^; 2) evidence for their alteration in PD is stronger than for other inflammatory biomarkers, as these three mediators were analyzed in the largest number of studies (34–44 for peripheral blood, 8–14 for CSF)^171^; 3) exercise consistently lowers TNF, IL-6, and CRP levels across multiple populations with conditions relevant to PD^173–176^. While TNF, IL-6, and CRP appear to be the most reliable markers for studying exercise-induced changes in inflammation in PD, other factors—including IL-1β, CX3CL1, and clusterin—are emerging as relevant to both PD and exercise. Figure 3 presents a model depicting the role of these inflammatory markers in PD and the potential effects of exercise.

### TNF

Evidence of elevated TNF protein levels or mRNA expression in the CSF, the striatum, and the substantia nigra of individuals with PD dates to 1994, marking the first reported cytokine alteration in PD^180,181^. Since then, extensive studies have examined TNF system dysregulation in both central and peripheral immune compartments of PD. Research indicates that innate and adaptive immune cells and microglia, produce higher TNF levels in individuals with PD compared to healthy controls^182^. Additionally, T lymphocytes in PD patients express higher TNF receptor levels, making them more susceptible to TNF’s biological effects^182^. Circulating TNF levels in both blood^183–185^ and CSF^183,186,187^ have consistently been reported as elevated in PD. Preclinical studies and epidemiological evidence suggests TNF plays a key role in PD pathogenesis^188,189^. In vitro and animal models of PD demonstrate TNF involvement in neuronal cell death^182^, while epidemiological studies reveal that early or mid-life exposure to anti-TNF therapy significantly reduces PD risk in individuals with IBD, a population already at elevated risk for PD^190,191^.

A recent meta-analysis by Khalafi et al. found that chronic aerobic exercise ( ≥ 7 weeks) significantly reduces soluble TNF levels in older adults^176^. Similarly, an earlier meta-analysis by Zheng et al. across five studies of chronic aerobic exercise ( ≥ 24 weeks) reported comparable reductions in blood TNF levels^192^. In individuals with PD, two small studies of moderate- and high-intensity interval training (8-12 weeks) have been shown to significantly lower soluble TNF levels in the blood^177,178^. Given that peripheral TNF alterations may reflect CNS changes in PD^171^, monitoring blood TNF levels in exercise trials could provide mechanistic insights into neuroinflammation while reducing reliance on invasive procedures.

### IL-6

The cytokine IL-6 has been extensively studied in PD, with evidence indicating significantly elevated levels of IL-6 in both blood^193,194^ and CSF^187,195^ in individuals with PD compared to controls^171^. Additionally, serum IL-6 levels inversely correlate with measures of functional mobility, gait speed, and Mini-Mental Status Examination scores^193,196^. While studies have shown significant reductions in peripheral blood IL-6 levels in response to chronic exercise in individuals with various chronic inflammatory conditions^173,174,176^, trials assessing the effects of chronic aerobic exercise on IL-6 levels in people with PD remain lacking. Unlike TNF, which has a well-established detrimental role in PD, the impact of IL-6 in PD and in response to exercise is more complex^197^. IL-6 exhibits multiple signaling modalities and cellular targets, acting as both a pro- and anti-inflammatory mediator^197,198^. Preclinical studies indicate that IL-6 can promote both neuronal cell death and survival after injury, and it is also necessary for mediating the beneficial effects of exercise^199^. Consequently, evaluating the mechanistic response to exercise using IL-6 is more challenging than with other cytokines, as it requires assessing various components of the IL-6 system, including soluble IL-6 receptor levels, in addition to IL-6 itself.

### CRP

As discussed in a recent perspective, the acute-phase protein CRP is the most studied biomarker of inflammation in PD and a key marker for evaluating the effects of exercise on inflammation^200^. A meta-analysis of 23 studies found that individuals with PD have significantly higher CRP levels both in the peripheral circulation and in the CSF compared to matched healthy controls^201^. Independent of disease duration or symptom severity, baseline CRP levels in PD participants are associated with an increased risk of death and poorer life prognosis^202^. Higher CRP levels also positively correlate with PD disease stage and motor symptoms^203,204^. In CSF, elevated CRP levels have also been linked to cognitive impairment, as well as increased severity of depression, anxiety, and fatigue in PD^205^. Multiple systematic reviews in middle-aged and older adults have shown that aerobic exercise, particularly at higher intensity and longer duration ( > 9 weeks), is associated with greater reductions in CRP levels^176,192,206^. However, no studies have yet examined the effects of aerobic exercise on CRP in individuals with PD. Although genetic data suggest that CRP is unlikely to play a direct role in PD pathogenesis^207^, CRP remains as a valuable biomarker for assessing overall inflammatory status in response to exercise. Unlike other mediators that are transiently expressed, CRP reflects the cumulative response of inflammatory cytokines and mediators. For further details, we refer the reader to our perspective^200^.

### IL-1β

The pro-inflammatory cytokine IL-1β is elevated in both peripheral blood and CSF in individuals with PD compared to controls^171^. Various pathways, including aggregated α-synuclein, activate the NLRP3 inflammasome, a multi-molecular complex that processes pro-IL-1β into the mature, bioactive form, leading to neuroinflammation and neuronal death by pyroptosis^208^. Inhibition of IL-1β or NLRP3 is protective in preclinical models of PD^208^; however, no human studies have been conducted to date. While meta-analyses do not show a consistent reduction in IL-1β levels in response to exercise, a sub-analysis indicates that combining aerobic and resistance training significantly lowers IL-1β in individuals with overweight/obesity and cardiometabolic diseases^209^. The effects of aerobic exercise alone on IL-1β remain less clear, with only one study in obese women reporting a decrease in blood IL-1β following three months of aerobic exercise^210^. The uncertainty regarding exercise’s effectiveness in lowering IL-1β may stem from challenges in accurately quantifying this cytokine in peripheral blood, posing an additional hurdle for future research.

### CX3CL1/Fractalkine

The chemokine CX3CL1 (fractalkine) has gained attention over the past decade as a biomarker of inflammation in PD and a potential link between PD and exercise^211^. A meta-analysis of 4 studies found a significant increase in CX3CL1 levels in peripheral blood of individuals with PD compared to controls, but no significant alterations in the CSF^171^. However, one study reported significantly reduced CSF levels in PD^212^. The effects of exercise on CX3CL1 remain inconclusive. CX3CL1 levels have been shown to decrease in young and middle-aged adults with mobility disabilities following 12 weeks of combined aerobic and resistance training^213^. Conversely, in a randomized controlled trial in adults with type 2 diabetes and coronary artery disease, a slight increase in circulating CX3CL1 levels was observed after 12 months of combined aerobic and resistance training^214^. In individuals with PD, 12 weeks of balance training significantly increased circulating CX3CL1 levels^215^, but the effects of aerobic exercise training remain unknown. The limited number of studies examining the impact of exercise on CX3CL1 levels, along with conflicting findings in both PD and exercise research, highlights the need for further investigation to determine the utility of CX3CL1 as a biomarker of inflammation in exercise interventions in PD.

### Clusterin

Clusterin is an emerging biomarker produced by hepatocytes in response to exercise and may play a role in modulating inflammation^142,216^. As an extracellular chaperone, clusterin exerts anti-inflammatory effects primarily by suppressing complement activation and preventing or slowing the aggregation of misfolded proteins, including α-synuclein, tau, and amyloid-beta^217,218^. De Miguel et al. demonstrated that plasma collected from voluntarily running mice and infused into sedentary mice reduces neuroinflammatory gene expression and brain inflammation^216^. However, immunodepletion of clusterin abolished these anti-inflammatory benefits^216^. Additionally, in a small cohort of veterans with MCI, six months of combined aerobic and resistance training led to increased plasma clusterin levels^216^. An exercise-induced increase in clusterin may be particularly relevant to PD, as studies have shown that exosomes isolated from the plasma of individuals with PD at Hoehn and Yahr stage II exhibit significantly lower clusterin expression compared to healthy individuals^219^. While further research is needed to confirm the effects of exercise on clusterin in PD, current evidence suggests that aerobic exercise may help increase clusterin levels, potentially offering neuroprotective benefits.

## Exercise-Induced Changes in Neuroendocrine Markers

Neuroendocrine abnormalities have been reported at all stages of PD and are associated with multiple motor and non-motor symptoms^220,221^. Mounting evidence suggests these abnormalities are an intrinsic feature of PD rather than a consequence of other physiological processes or medication side effects. The neuroendocrine system integrates endocrine outputs from the nervous system and peripheral hormones that influence brain function. Neuroendocrine changes in PD affect various physiological functions, including stress response (cortisol), circadian rhythm (melatonin), insulin resistance (insulin), bone metabolism (vitamin D), and aging (klotho). Disruptions in these pathways or altered hormone levels have been linked to increased risk of PD. For example, epidemiological studies associate increased stress^222,223^, circadian rhythm dysfunction^224,225^, type 2 diabetes^226,227^, vitamin D deficiency^228–230^, and aging^231^ with a higher risk of developing PD.

Most neuroendocrine abnormalities in PD have primarily been identified in blood or saliva, with fewer studies assessing CSF. Cortisol levels are consistently elevated in blood^232–234^ and saliva^235,236^ of individuals with PD. Conversely, melatonin, insulin, and vitamin D levels are typically reduced in PD, though CSF studies for these markers remain limited^237–240^. Klotho, a longevity-related hormone, is significantly lower in the CSF of individuals with PD compared to healthy controls, but findings in blood are inconsistent^241^. While Kakar et al. reported no change in plasma klotho levels in moderate to advanced PD^242^, Sanscessario et al. found lower serum klotho levels in early-stage PD^243^. These findings suggest that hormone levels may vary with disease stage and may reflect distinct peripheral and CNS pools.

**Fig. 4:**
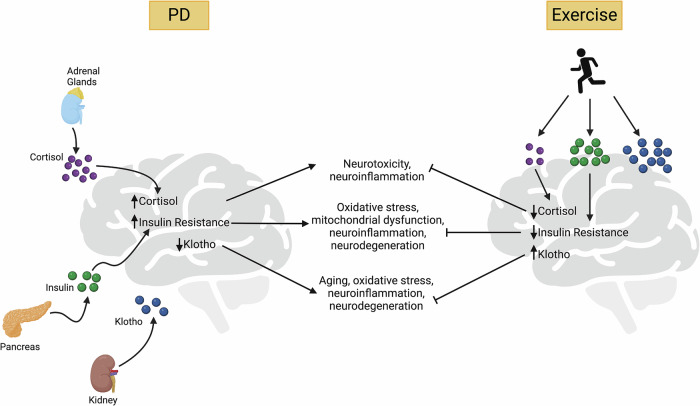
Exercise-induced changes in neuroendocrine markers. In PD, there is persistent elevation of stress hormone cortisol, increased insulin resistance, and reduction in klotho levels. These abnormalities have been linked to increased neuroinflammation, oxidative stress, neurodegeneration, and aging. Chronic aerobic exercise can help lower blood cortisol levels, reduce insulin resistance, and increase blood klotho levels, which may counter these pathological pathways. While cortisol and insulin can cross the BBB, klotho cannot cross the BBB and has two distinct pools (CNS and periphery). PD Parkinson’s disease, BBB blood brain barrier, CNS central nervous system. Created in BioRender. Mehta, N. (2025) https://BioRender.com/j77o278.

Exercise has the potential to modify the neuroendocrine abnormalities associated with PD. Chronic exercise training reduces hormonal stress responses, lowering cortisol levels^108,244^. It also enhances melatonin secretion^245,246^, improves insulin sensitivity both peripherally and in the CNS^247,248^, and increases circulating vitamin D^249^ and klotho levels^250–252^. Among these neuroendocrine markers, cortisol, insulin, and klotho may serve as robust biomarkers for assessing exercise benefits in PD because: 1) they exhibit consistent abnormalities in PD; 2) they play key roles in PD-related pathophysiological pathways; and 3) they have been shown to respond significantly to exercise (Fig. 4).

## Cortisol

Cortisol has widespread effects on the brain, affecting mood, behavior, cognition, and stress response regulation^253^. Stress modulates motor system function, as most parts of the motor regions express glucocorticoid receptors, the primary targets for cortisol^254^. In PD mouse models, chronic stress exacerbates motor deficits, accelerates nigrostriatal neurodegeneration, and impairs compensatory motor recovery^255^. In individuals with PD, cortisol levels have been shown to correlate with severity of depression, prevalence of anxiety and anhedonia, longer sleep latency, and increased risk-taking behavior^236,254,256,257^. However, the relationship between cortisol and PD motor symptoms remains inconsistent. While Haglin et al. found higher cortisol levels correlated with greater motor symptom severity (as measured by MDS-UPDRS motor scores)^258^, Muller and Muhlack reported significantly lower cortisol levels in individuals at advanced PD stages compared to those in earlier stages^259^. Interestingly, acute levodopa administration has been shown to decrease cortisol levels, correlating with motor symptom improvement, suggesting that interventions lowering cortisol may help alleviate PD symptoms^259^.

Chronic exercise training induces neuroendocrine adaptations that ultimately reduce cortisol levels^260^. In women with MCI, 6 months of high-intensity aerobic exercise reduced plasma cortisol levels and improved executive cognitive function^108^. A meta-analysis by Beserra et al. found that regular exercise reduces daytime cortisol levels in individuals with major depressive disorder, with aerobic and more frequent exercise yielding greater effects^244^. While research on exercise and cortisol regulation in PD is limited, Smyth et al. demonstrated that high-intensity treadmill training reduced cortisol secretion during the post-awakening period in individuals with PD^261^. These findings suggest that cortisol may serve as a promising biomarker for assessing exercise response and its impact on PD symptoms.

### Insulin

Insulin plays a central role in peripheral glucose metabolism, but within the central nervous system, it also regulates dopaminergic transmission, maintenance of synapses, synaptic plasticity, and neuronal survival and growth^262^. In PD, both insulin deficiency and insulin resistance contribute to impaired brain insulin signaling which may drive neuroinflammation, mitochondrial dysfunction, and oxidative stress^237,263–266^. In rodent models, insulin resistance has been linked to reduced surface expression of dopamine transporters in the striatum, increased ROS, and α-synuclein aberrant expression^267–270^. Induction of insulin signaling with IGF-1 and reversal of insulin resistance has been shown to suppress α-synuclein aggregation and toxicity in cultured cells^271^. Insulin resistance in individuals with PD associates with a more severe phenotype, accelerated disease progression, and increased risk of cognitive decline, whereas individuals treated with antidiabetic drugs have a lower risk of developing PD^272^. Several antidiabetic drugs, i.e. exenatide and lixisenatide, are being explored as potential disease-modifying agents for PD^273,274^.

Exercise is well known to improve insulin sensitivity peripherally^248,275^, and has more recently been shown to positively modulate brain insulin signaling pathways^276,277^. In rodent models of memory impairment, exercise improves insulin signaling alongside cognitive function^278^. In middle-aged sedentary individuals, exercise for 8 weeks increases brain insulin sensitivity following intranasal insulin administration^279^. Exercise also upregulates IGF-1 gene expression and protein levels in several brain regions, especially those involved in learning and cognition^280^. Additionally, it increases circulating IGF-1 in the periphery, which can cross the BBB and reach the brain^281^. Given the role of insulin resistance in PD pathogenesis and the ability of exercise to mitigate it, insulin may serve as a valuable biomarker for assessing exercise responsiveness in individuals with PD.

### Klotho

α-Klotho (hereafter referred to as klotho) is a pleiotropic protein recognized as a master regulator of aging. In mice, klotho deficiency leads to a significantly shortened lifespan and premature aging, while its overexpression extends lifespan by approximately 30%^282,283^. Klotho functions both as a membrane-bound coreceptor for fibroblast growth factor 23 and as a circulatory endocrine mediator exhibiting anti-inflammatory, antioxidant, and neuroprotective properties^284,285^. Klotho inhibits multiple aging-related pathways, including transforming growth factor β, IGF-1, Wnt and NF-κB^282,286–288^. In the context of PD, klotho overexpression or exogenous administration protects dopaminergic neurons against oxidative injury and alleviates astrogliosis, apoptosis, and oxidative stress^289,290^. Additionally, it improves cognition, motor function, and synaptic plasticity in PD mouse models^289^. In humans, higher klotho levels have been associated with increased lifespan and enhanced cognition in aging populations^291–293^. Furthermore, elevated klotho levels correlate with reduced amyloid-beta burden and improved cognition in individuals at risk for Alzheimer’s disease (AD)^294^.

Recent studies have linked klotho to PD progression. CSF klotho levels have been shown to be lower in individuals with moderate stage PD compared to controls, correlating with greater motor impairment, higher Hoehn and Yahr disability stages, and poorer cognitive performance^241,295,296^. Plasma klotho levels appear unchanged in moderately advanced PD, although women with PD exhibit higher levels than men^242^. Interestingly, one study found the opposite pattern in early stages of PD, with higher CSF levels of klotho and lower serum klotho levels^243^. These discrepancies may stem from differences in assay methodologies or disease stage. Like IGF-1 dynamics, an initial compensatory increase in CSF klotho may occur in early PD but decline as the disease progresses^103–106^.

Beyond neuroprotection, klotho is associated with better lower extremity strength and physical function in older adults^297,298^. Higher klotho levels have also been observed in physically trained individuals compared to their sedentary counterparts^299^. Chronic aerobic training increases plasma klotho levels in healthy middle-aged adults and older women^250,251,300^. A recent meta-analysis confirmed that at least 12 weeks of aerobic exercise significantly elevates circulating klotho levels in both healthy individuals and those with chronic diseases^252^. However, no studies have yet examined how exercise influences klotho levels in individuals with PD. Mechanistically, exercise may upregulate klotho via myokines such as irisin. Jin et al. demonstrated that irisin enhances cognition and reduces mortality in mice following cerebral ischemia through klotho upregulation^301^. Notably, these benefits were abolished in klotho-knockout mice, underscoring klotho’s critical role in exercise-mediated neuroprotection. Future research should explore how klotho responds to exercise in people with PD and whether its modulation correlates with symptom progression and disease severity.

## Exercise-Induced Changes in Markers of PD Pathology

### α-Synuclein

Exercise is likely to affect PD pathogenesis and risk. Among candidate biomarkers of PD pathology, α-synuclein is a key protein, as it constitutes the major component of Lewy bodies, the pathological hallmark of PD^302^. α -Synuclein is highly conserved within mammals and widely expressed in the brain and throughout the body, including the myocardium, skin fibroblasts, saliva, muscle, bone marrow, liver, and spleen^303^. It can cross the BBB bidirectionally, moving between the blood and the brain. In the brain, α-synuclein plays a crucial role at presynaptic sites, where it regulates neurotransmitter release and reuptake, synaptic vesicle trafficking, and exerts chaperone-like activity^304^. In PD, α-synuclein misfolds into oligomers and amyloid fibrils, adopting a β-sheet conformation.

α-Synuclein centrality to PD is further underscored by mutations in the SNCA gene, which cause rare familial forms of PD^305,306^, and genetic variants of α-synuclein that increase the risk of sporadic PD^307^. Although total α-synuclein levels in the CSF are slightly lower in PD participants compared to controls, values largely overlap at the population level^308^. A major recent breakthrough in PD diagnostics is the α-synuclein seed amplification assay (SAA), also known as real-time quaking-induced conversion (RT-QuIC) or protein misfolding cyclic amplification assay (PMCA). This assay detects misfolded α-synuclein with high diagnostic accuracy (88-97% sensitivity and 90-99% specificity) when performed on CSF^309–321^.

Notably, studies of prodromal PD also show a high positivity rate for α-synuclein SAA^315,319–321^, indicating that misfolded α-synuclein can be detected decades before clinical diagnosis, even before dopaminergic neurodegeneration becomes apparent on brain imaging^320^. To facilitate wider clinical application, α-synuclein SAA is being explored in more accessible biofluids and tissues, such as skin biopsies^322–324^, olfactory epithelium^325^, saliva^326,327^, blood extracellular vesicles^328^ and in serum^329^. While initial results, especially from skin biopsies, are promising, the reliability and accuracy of SAA using these peripheral sources require further validation through independent studies.

**Fig. 5:**
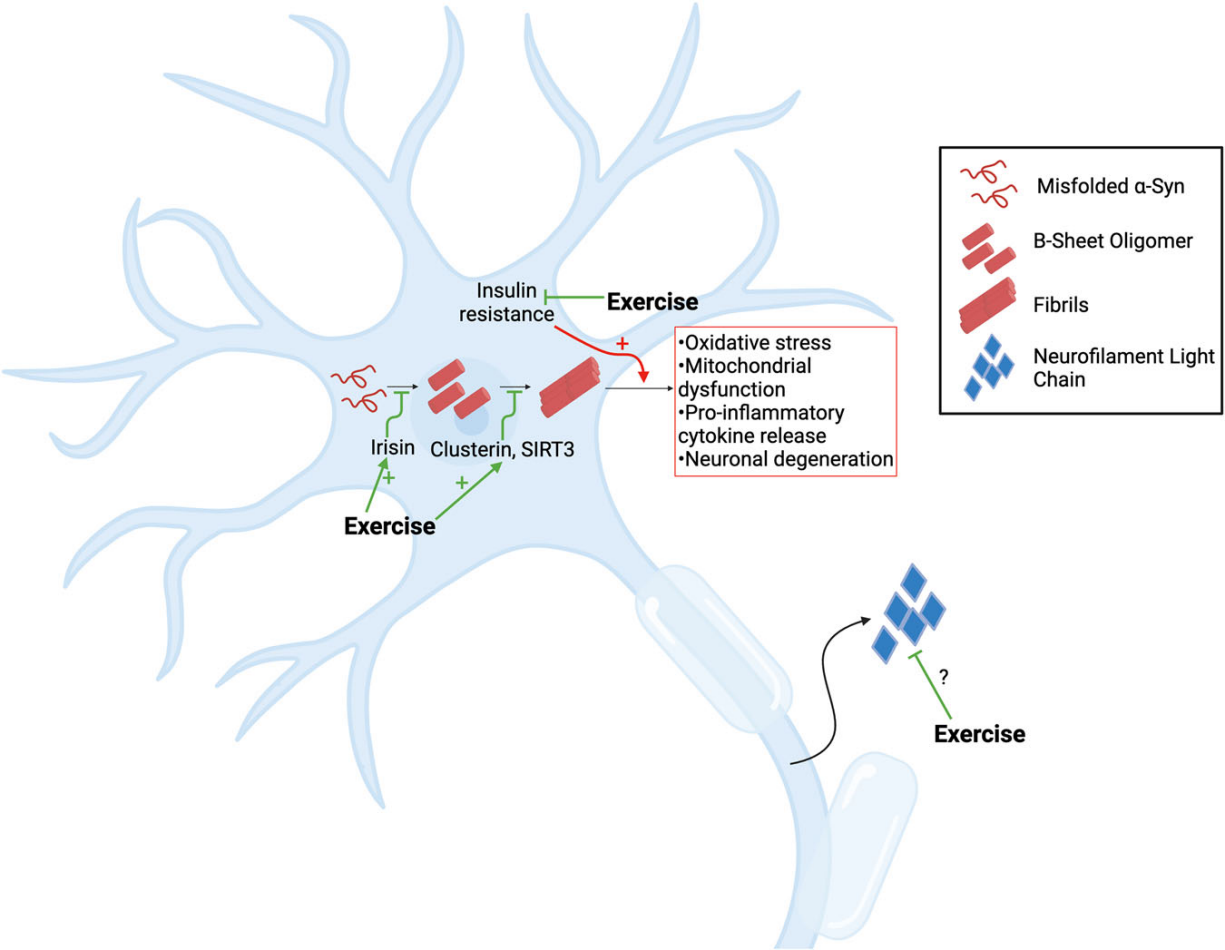
Potential interactions between exercise and markers of PD pathology. Exercise may induce increases in irisin, clusterin, and SIRT3, markers that have been shown to reduce conversion of misfolded **α**- synuclein to pathological **α**-synuclein. Pathological **α**-synuclein aggregate formation in PD can lead to oxidative stress, mitochondrial dysfunction, pro-inflammatory cytokine release, and neuronal degeneration. Insulin resistance also associates with increased **α**-synuclein aggregate formation, but exercise may decrease insulin resistance and thus reduce aggregated protein. Axonal injury in PD releases NfL with preliminary evidence suggesting exercise may lower circulating NfL levels. α-syn, α-synuclein; SIRT3 sirtuin-3, NfL neurofilament light chain. Created in BioRender. Mehta, N. (2025) https://BioRender.com/h90y253.

Although clinical evidence linking exercise and α-synuclein is currently lacking, preclinical studies provide insight into this relationship. In rodent models of PD, mild- to moderate-intensity treadmill exercise (5 days a week for 6-8 weeks) has been associated with reduced α-synuclein levels in striatum and substantia nigra, alongside improvements in motor function^330–334^. As clinical research advances, future studies will help elucidate how exercise influences α-synuclein pathology in PD and how to measure its potential changes with quantitative assays. Figure 5 proposes a model illustrating how exercise-induced biomarkers may modulate pathological α-synuclein.

### Neurofilament light chain

Neurofilament light chain (NfL) is a promising biomarker of disease severity and progression in PD. However, it lacks specificity, as elevated NfL levels are observed in multiple neurodegenerative disorders^335,336^. NfL is a structural component of the neuronal cytoskeleton, released into biofluids following axonal damage in neurodegenerative disorders, neuroinflammatory conditions, traumatic nervous system injury, stroke, and during normal aging. Serum or plasma NfL correlate with CSF levels^337,338^ making it a more accessible biomarker than CSF sampling. Despite its nonspecific nature, NfL shows potential for monitoring disease progression, prognosis, and treatment response^338^. Whether NfL can serve as an exercise response biomarker remains unclear due to limited research. In MS, an 8-week aerobic training program lowered serum NfL levels^339^, whereas a 16-week aerobic training program in mild AD patients showed no significant changes^340^. In a toxin-induced hemiparkinsonian rat model, moderate-intensity aerobic exercise reduced serum NfL levels^341^. To date, no clinical studies have examined NfL changes in response to exercise in individuals with PD. Further studies are needed to determine whether exercise influences NfL levels in PD and if it could serve as a reliable marker for neuroprotection or disease modification.

## Exercise-Induced Changes in Other Diseases of the Nervous System

The findings summarized in this review show striking similarities to those observed in other diseases of the central nervous system. In AD, large-scale transcriptomic analyses have identified exercise and physical activity as the top theoretical treatments, reversing the expression patterns of hundreds of AD-related genes across multiple functional categories^342^. While similar gene expression analyses have not been conducted in PD, comparable outcomes are expected. A review by Paillard, Blain and Bernard examined the effects of exercise in AD, similar to the approach taken in this paper^343^. They concluded that aerobic exercise promotes angiogenesis and the release of neurotrophic factors (e.g., IGF-1, BDNF, VEGF), which enhance cerebral blood flow and support neuroplasticity. Additionally, IGF-1 is believed to facilitate amyloid-beta clearance and reduce tau hyperphosphorylation, both of which are central to AD pathology. Exercise has also been shown to provide significant benefits in MS^344^. However, unlike PD and AD, no fluid biomarkers have yet emerged as clear indicators of exercise-induced therapeutic effects in MS^344^.

## Conclusion

Chronic aerobic exercise induces multidimensional changes by modulating various pathways involved in the development and progression of PD. In this review, we have identified BDNF, GDNF, IGF-1, VEGF, TNF, IL-6, CRP, cortisol, insulin, and klotho as candidate biomarkers with the strongest evidence for their responsiveness to aerobic exercise and potential roles in neuroprotection and symptom alleviation in PD. Additionally, emerging evidence suggests that irisin, GPLD1, SIRT3, IL-1β, CX3CL1, and clusterin may also play significant roles in PD and exercise adaptation. Table 1 summarizes all biomarkers. Currently, there is limited data on how exercise affects markers of PD pathology, including α-synuclein and NfL, particularly in the prodromal and early disease stages. Future studies should explore whether exercise influences these biomarkers and their relationship with disease progression.

**Table 1:** Biofluid Markers Relevant to Parkinson’s Disease and Exercise

Biomarker	Role in Neurodegenerative Disease	Source	Crosses BBB?	Direction of change with PD^1^	Direction of change with chronic aerobic exercise^2^ (Level of Evidence)
***Neurotrophic/Neuroprotective Markers***					
**BDNF**	Neurogenesis, synaptic plasticity, neuroprotection	Brain, muscle	Yes	**↓** levels (blood^57,60^, brain^69^)	**↑** levels (blood) in healthy adults^78–80^ (Level I) and people with PD^80–82^(Level I)
**GDNF**	Neuroprotection	Brain, muscle	No	**↓** levels (blood^59,83^, brain^54^)	**↑** levels (blood) in healthy adults^97^ (Level IV)
**IGF-1**	Neurogenesis, angiogenesis, synaptic plasticity	Liver, brain	Yes	**↑** levels (blood^61,103–106^, CSF^61^)	**↑** levels (blood) in elderly men^108^ (Level II) and in postmenopausal women^109^ (Level I)
**VEGF**	Neurogenesis, angiogenesis	Brain, muscle	Yes	No change in levels (blood^119^), **↑** levels (CSF^56^)	**↑** levels (blood) in older adults^126^ (Level I)
**Irisin**	Neuroprotection, increases BDNF levels	Brain, muscle	Yes	**↓** levels (blood)^137^	**↑** levels (blood) in healthy adults and patients with chronic disease^138^ (Level I) and people with PD^134^ (Level IV)
**GPLD1**	Neurogenesis, increases BDNF levels	Liver	No	Unknown	**↑** levels (blood) in active older adults^142^ (Level IV)
**SIRT3**	Counters mitochondrial dysfunction and oxidative stress, decreases α-synuclein oligomer formation	Brain, liver, muscle, heart, kidney	Modulates BBB permeability	**↓** levels (SNc^150^)	**↑** levels (blood, muscle) in healthy adults^156^ (Level IV) and overweight adolescents^156,157^ (Level IV)
**Lactate**	Neuronal metabolism; increases BDNF levels; anti-inflammatory effects	Brain, muscle	Yes	**↑** levels (CSF^163^) in early PD; no change (serum) in moderately advanced PD^164^	**↓** levels (blood) in people with MS^167^ and type 2 DM^168^ (Level I) and in people with PD^166^ (Level II)
NGF	Neuroprotection, synaptic plasticity	Brain	No	**↓** levels (blood^58^, brain^345^)	Inconclusive^346^
CDNF	Neuroprotection, anti-inflammatory effects, modulate protein homeostasis	Brain	No	**↑** levels (brain^54^), unchanged (blood^62^)	No human studies
MANF	Neuroprotection, anti-inflammatory effects, modulate protein homeostasis	Brain	No	**↑** levels (blood^62^)	Unknown
***Inflammatory Markers***					
**TNF**	Pro-inflammatory cytokine, immunostimulation, induces apoptosis	Brain, muscle, skin	Yes	**↑** levels (blood^171,184,185^, CSF^171,186,347^, brain^181,186^)	**↓** levels (blood) in older adults^176,192^ (Level I), people with type 2 DM^173,174^ (Level 1), overweight adults^209^ (Level 1), and in people with PD^177,178^ (Level II)
**IL-6**	Pro-inflammatory cytokine, immunostimulation, induces coagulation	Brain, muscle skin, liver	Yes	**↑** levels (blood^171,185,187,193,194,347^, CSF^187,195,347^)	**↓** levels (blood) in older adults^192^ (Level I), people with type 2 DM^173,174^ (Level I), overweight adults^209^ (Level 1)
**CRP**	Pro-inflammatory cytokine, immunostimulation, induces cytokine release	Liver, lung	No, can modulate BBB permeability	**↑** levels (blood^171,201^, CSF^171,201^)	**↓** levels (blood) in older adults^176,192^ (Level I), people with type 2 DM^173,174^ (Level 1), overweight adults^209^ (Level 1)
**IL-1β**	Pro-inflammatory cytokine, immunostimulation, promotes tumor growth	Brain, lymphoid organs, liver, lung, GI tract	Yes	**↑** levels (blood^171,184,187^, CSF^171,187^)	**↓** levels (blood) in overweight adults and adults with cardiometabolic diseases^210^ (Level I for combined training) and in obese women^209^ (Level II)
**CX3CL1** (Fractalkine)	Pro-inflammatory chemokine, microglia activation	Brain, colon, heart, lung, liver, kidneys	No, can modulate BBB permeability	**↑** levels (blood^171,348^)	Variable results^213,214^
**Clusterin**	Extracellular chaperone with anti-inflammatory effects; prevents or slows the formation of amorphous aggregates and fibrils	Brain, kidney, heart, liver	Yes	**↓** levels (blood^219^)	Increased levels (blood) in adults with MCI^216^ (Level IV for combined training)
CCL2 (MCP1)	Pro-inflammatory chemokine, immunostimulation, promotes tumor growth	Brain, widespread in tissues	Yes	**↑** levels (blood^171,184^, CSF^171,347^)	**↓** levels (blood) in adults with metabolic syndrome^349,350^ (Level II for combined training)
CXCL12	Pro-inflammatory chemokine, immunostimulation, promotes angiogenesis	Brain, widespread in tissues	Yes	**↑** levels (blood^348,351^)	**↓** levels (blood) in cancer survivors^350^ (Level III)
sTNFR1	Pro-inflammatory effects, receptor for TNF	Brain, widespread in tissues	No, can modulate BBB permeability	**↑** levels (blood^171^)	**↓** levels (blood) in healthy women^352^ (Level IV)
NT-proBNP	Anti-inflammatory effects, increases in response to inflammation	Heart, brain	No	**↑** levels (blood^171,353^)	**↓** levels (blood) in healthy adults^354^ (Level III) and patients with heart failure^355^ (Level I)
IFN-α2	Anti-inflammatory cytokine, induces NO production	Brain, widespread in tissues	Minimally	**↓** levels (blood^171^)	No human studies
IL-4	Anti-inflammatory cytokine, microglia homeostasis	Brain, widespread in tissues	No	**↓** levels (blood^171,185^)	No Δ (blood) in middle-aged and older adults^192^ (Level I) and no Δ in women^356^ (Level II)
Nitric oxide	Anti-inflammatory under physiological conditions, pro-inflammatory mediator under abnormal conditions	Brain, endothelial cells	Yes	**↑** levels (blood^357^, CSF^171,358^)	**↑** levels (blood) in young, middle-aged and older adults^359^ (Level I)
***Neuroendocrine Markers***					
**Cortisol**	Stress response, pro-inflammatory effect and increased insulin resistance under chronic stress	Adrenal glands	Yes	**↑** levels (blood^232–234^, saliva^235,236^)	**↓** levels (blood, saliva) in adults with depression^244^ and women with MCI^108^ (Level I) and people with PD^261^ (Level IV)
**Klotho**	Longevity, counters oxidative stress and inflammation, regulates vitamin D metabolism	Brain, kidney	No	**↑** levels (CSF^243^), **↓** levels (blood^243^) in early stage; **↓** levels (CSF^241^), unchanged levels (blood^242^) in moderate stage	**↑** levels (blood) in healthy adults and adults with chronic disease^252^ (Level I)
**Insulin**	Regulates dopaminergic transmission, maintenance of synapses, synaptic plasticity, and neuronal survival and growth	Brain, pancreas	Yes	**↓** levels (blood^237^), **↑** insulin resistance^266^	**↑** insulin sensitivity in healthy adults^248,275^ (Level I)
Vitamin D	Counters inflammation and oxidative stress, transcriptional regulation of neurotrophins	Brain, skin	Yes	**↓** levels (blood^238,239^)	**↑** levels (blood) in healthy adults and adults with chronic disease^249^ (Level I)
Melatonin	Counters neuronal loss and oxidative stress, inhibits formation of α-synuclein aggregates	Pineal gland	Yes	**↓** levels (blood^240^)	**↑** levels (blood) in middle-aged men^246^ (Level IV)
***Markers of PD Pathology***					
**α-synuclein**	Neuronal degeneration, synaptic dysfunction	Brain, GI tract, heart, skin	Yes	**↑** misfolded forms (CSF^312,314,317–321,360^)	No human studies
**NfL**	Cytoskeletal component, released in setting of neuronal damage and inflammation	Brain	Yes	**↑** levels (blood^335^, CSF^335^)	Variable in people with MS^339^ and AD^340^

**↑** indicates increase**; ↓** indicates decrease. ^1^Refer to Supplemental Table 1A for details on magnitude of change in PD for each biomarker. ^2^Refer to Supplemental Table 1B for details on magnitude of change in response to aerobic exercise for each biomarker.

*Level of evidence based on hierarchy of evidence-based medicine as described by Stillwell et al.^361^. Level I (evidence from systematic review or randomized-controlled trials or meta-analysis, Level II (evidence from at least one well-designed RCT, Level III (evidence from well-designed controlled trial without randomization, Level IV (evidence from well-designed case control or cohort study, Level V (evidence from systematic review of descriptive/qualitative studies, Level VI (evidence from single descriptive or qualitative study, Level VII (expert opinion).

*BBB* blood-brain barrier. *PD* Parkinson’s disease, *AE* aerobic exercise, *CSF* cerebrospinal fluid, *BDNF* brain-derived neurotrophic factor, *GDNF* glial cell line-derived neurotrophic factor, *IGF-1* insulin-like growth factor 1, *VEGF* vascular endothelial growth factor, *NGF* nerve growth factor, *CDNF* cerebral dopamine neurotrophic factor, *MANF* mesencephalic astrocyte-derived neurotrophic factor, *GPLD1* glycosylphosphatidylinositol-specific phospholipase D1, *TNF* tumor necrosis factor, *CRP* C-reactive protein, *sTNFR1* soluble TNF receptor-1, *NT-proBNP* N-terminal pro-B-type natriuretic peptide, *IL* interleukin, *IFN* interferon.

Many of the biomarkers identified likely mediate bidirectional crosstalk between the periphery and the brain, driving exercise-induced neuroprotection. Tracking these biomarkers in exercise intervention studies will enhance our mechanistic understanding of how chronic aerobic exercise benefits individuals with PD. Furthermore, identifying a biofluid profile that reliably responds to exercise would facilitate comparisons between different exercise intensities and modalities to determine optimal dosage effects. One such study, SPARX3, aims to evaluate the effects of two different aerobic exercise intensities on these biomarkers in people with early-stage PD^44^. It will also be essential to analyze associations between specific biomarkers and motor/non-motor PD symptoms as well as disease progression. Once a validated panel of exercise response biomarkers is established, these markers could potentially be used in clinical settings to track an individual’s response to exercise and tailor regimens to elicit optimal molecular responses for symptom management or disease modification. However, factors such as sensitivity/specificity, cost, assay variability, and feasibility of routine biomarker testing must be considered for clinical application.

Our review has several limitations: 1) Biomarker selection is based on a literature review of markers implicated in PD and linked to exercise but may not include all responsive markers; 2) it focuses on aerobic exercise without comparing effects across different exercise modalities (e.g., resistance training); and 3) the studies reviewed vary in quality and sample sizes (see Supplementary Information), limiting generalizability. While this review primarily addresses chronic aerobic exercise, future research should also investigate acute exercise effects on these biomarkers. For instance, lactate, cortisol, and inflammatory markers typically increase immediately after acute exercise, contrasting with their long-term reductions observed with chronic exercise. Understanding how acute vs. chronic biomarker changes impact PD symptoms and disease mechanisms will be crucial for optimizing exercise-based interventions.

## Supplementary information

Supplementary Material
