# Supplementary material for: Aerobic exercise-induced changes in fluid biomarkers in Parkinson’s disease
Source: NPJ Parkinsons Dis. 2025 Jul 1;11:190. doi: 10.1038/s41531-025-01042-8 (PMC12215721; doi:10.1038/s41531-025-01042-8)
Supplement: Supplementary file 1 — Supplementary Material [file 41531_2025_1042_MOESM1_ESM.pdf]

Supplemental Table 1A. Biomarkers Changes in Parkinson's Disease

Biomarker	Sample Source	Study (reference number)	Type of Study	Sample size	Direction of Change in PD	Magnitude of Change	Associated P-value
Neurotrophic/Neuroprotective Markers							
BDNF	Serum	Jiang et al. 2019 (#57)	Meta-analysis	838 PD, 657 HC (9 studies)	Decreased	SMD = -1.03	p = 0.012
BDNF	Serum	Rahmani et al. 2019 (#60)	Meta-analysis	434 PD, 545 HC (8 studies)	Decreased	MD = -2.99 ng/mL	p = 0.03
BDNF	Brain tissue	Howells et al. 2000 (#69)	Original research	5 PD, 5 HC	Decreased	30% reduction in people with PD	p = 0.001
GDNF	Serum	Tong et al. 2023 (#59)	Original research	105 PD, 45 HC	Decreased	17% reduction in people with PD	p = 0.008
GDNF	Serum	Liu et al. 2020 (#83)	Original research	112 PD, 43 HC	Decreased	21% reduction in people with PD	p < 0.05
GDNF	Brain tissue	Virachit et al. 2019 (#54)	Original research	10 PD, 9 HC	Decreased	19% reduction in hippocampus of people with PD	p = 0.04
IGF-1	Blood	Castilla-Cortazar et al. 2020 (#103)	Systematic review	949 PD and HC (11 studies)	Increased	2-54% elevation in people with PD	p <0.01 for most studies
IGF-1	Serum	Shi et al. 2023 (#104)	Original research	100 PD, 100 HC	Increased	51% elevation in people with PD	p < 0.001
IGF-1	Serum	Picillo et al. 2017 (#105)	Original research	405 PD, 191 HC	Unchanged	None	n/a
IGF-1	Serum	Godau et al. 2010 (#106)	Original research	12 PD, 12 HC	Increased	49% elevation in people with PD	p < 0.001
IGF-1	Serum	Mashayekhi et al. 2010 (#61)	Original research	38 PD, 38 HC	Increased	54% elevation in people with PD	p < 0.001
IGF-1	CSF	Mashayekhi et al. 2010 (#61)	Original research	38 PD, 38 HC	Increased	36% reduction in people with PD	p < 0.001
VEGF	Serum	Infante et al. 2007 (#119)	Original research	191 PD, 121 HC	Unchanged	None	n/a
VEGF	CSF	Janelidze et al. 2015 (#56)	Original research	82 PD, 38 HC	Increased	10% elevation in people with PD	p = 0.012

Irisin	Plasma	Shi et al. 2024 (#137)	Original research	100 PD, 70 HC	Decreased	5% reduction in people with PD	$p = 0.021$
SIRT3	Brain tissue	Trinh et al. 2023 (#150)	Original research	8-11 PD and HC	Decreased	57% reduction in SNc, 34% reduction in hippocampus in people with PD	$p < 0.05$
Lactate	CSF	Liguori et al. 2022 (#163)	Original research	101 de novo PD, 60 HC	Increased	11% elevation in people with PD	$p = 0.03$
Lactate	Serum	Miyaue et al. 2020 (#164)	Original research	20 PD, 18 HC	Unchanged	None	n/a
NGF	Serum	Lorigados Pedre et al. 2002 (#58)	Original research	17 PD, 16 HC	Decreased	78% reduction in people with PD	$p < 0.01$
NGF	Brain tissue	Mogi et al. 1999 (#345)	Original research	19 PD, 19 HC	Decreased	97% reduction in substantia nigra in people with PD	$p < 0.05$
CDNF	Brain tissue	Virachit et al. 2019 (#54)	Original research	10 PD, 9 HC	Increased	41% elevation in hippocampus in people with PD	$p = 0.02$
CDNF	Serum	Galli et al. 2019 (#62)	Original research	34 PD, 35 HC	No difference	21% elevation in people with PD	$p = 0.25$
MANF	Serum	Galli et al. 2019 (#62)	Original research	34 PD, 35 HC	Increased	104% elevation in people with PD	$p < 0.001$
Inflammatory Markers							
TNF	Blood and CSF	Qu et al., 2023 (#171)	Meta-analysis	9,032 PD, 12,628 HC (152 studies)	Increased	Hedges' g = 0.593	$p < 0.001$
TNF	Blood	Reale et al., 2009 (#184)	Original research	40 PD, 40 HC	Increased	192% elevation in people with PD	$p < 0.001$
TNF	Serum	Brodacki et al., 2008 (#185)	Original research	55 PD, 20 HC	Increased	17% elevation in people with PD (change inferred from figure)	$p < 0.001$
TNF	CSF	Mogi et al., 1994 (#186)	Original research	15 PD, 16 HC	Increased	332% elevation in people with PD	$p < 0.005$
TNF	Blood and CSF	Schröder et al., 2018 (#347)	Original research	10 PD, 13 HC	Increased	Significant elevation in CSF, exact value not reported	$p < 0.05$
TNF	Brain tissue	Boka et al., 1994 (#181)	Original research	3 PD, 3 HC	Increased	7-fold elevation in people with PD	not reported

TNF	CSF and Brain tissue	Mogi et al., 1994 (#186)	Original research	9 PD, 8 HC	Increased	266% elevation in striatum in people with PD	$p < 0.05$
IL-6	Blood and CSF	Qu et al., 2023 (#171)	Meta-analysis	9,032 PD, 12,628 HC (152 studies)	Increased	Hedges' g = 0.603	$P < 0.001$
IL-6	Serum	Brodacki et al., 2008 (#185)	Original research	55 PD, 20 HC	Increased	43% elevation in people with PD (change inferred from figure)	$p < 0.001$
IL-6	Plasma and CSF	Blum-Degen et al., 1995 (#187)	Original research	22 PD, 12 HC	Increased	50% elevation in CSF in people with PD (change inferred from figure)	$P = 0.013$
IL-6	Serum	Scalzo et al., 2010 (#193)	Original research	44 PD, 22 HC	Increased	57% elevation in people with PD	$P = 0.01$
IL-6	Blood	Diaz et al., 2022 (#194)	Original research	22 PD, 14 HC	Increased	83% elevation in people with PD (change inferred from figure)	$p = 0.005$
IL-6	CSF	Müller et al., 1998 (#195)	Original research	22 PD	Increased	62% elevation in people with PD	$p = 0.013$
IL-6	Blood and CSF	Schröder et al., 2018 (#347)	Original research	10 PD, 13 HC	Increased	Significant elevation in CSF, exact value not reported	$p < 0.05$
CRP	Blood and CSF	Qu et al., 2023 (#171)	Meta-analysis	9,032 PD, 12,628 HC (152 studies)	Increased	Hedges' g = 0.510	$p < 0.001$
CRP	Serum and CSF	Qiu et al., 2019 (#201)	Meta-analysis	2646 PD, 193 HC (23 studies)	Increased	Serum SMD = 1.115; CSF SMD = 1.127	$p < 0.001$
IL-1β	Serum and CSF	Qu et al., 2023 (#171)	Meta-analysis	9,032 PD, 12,628 HC (152 studies)	Increased	Hedges' g = 1.3	$p < 0.001$
IL-1β	Blood	Reale et al., 2009 (#184)	Original research	40 PD, 40 HC	Increased	45% elevation in people with PD	$p < 0.001$
IL-1β	Plasma and CSF	Blum-Degen et al., 1995 (#187)	Original research	22 PD, 12 HC	Increased	29% elevation in CSF in people with PD (change inferred from figure)	$P = 0.021$
CX3CL1	Serum and CSF	Qu et al., 2023 (#171)	Meta-analysis	9,032 PD, 12,628 HC (152 studies)	Increased	Hedges' g = 0.361	$p < 0.001$

CX3CL1	Plasma	Li et al., 2022 (#348)	Original research	76 PD, 76 HC	Increased	86% elevation in people with PD	$p = 0.006$
Clusterin	Plasma	Kitamura et al., 2018 (#219)	Original research	16 PD, 8 HC	Decreased	0.5-0.6- fold reduction in people with PD	$p < 0.05$
CCL2/MCP1	Serum and CSF	Qu et al., 2023 (#171)	Meta-analysis	9,032 PD, 12,628 HC (152 studies)	Increased	Hedges' g = 0.911	$p = 0.007$
CCL2/MCP1	Blood	Reale et al., 2009 (#184)	Original research	40 PD, 40 HC	Increased	228% elevation in PD	$p < 0.001$
CCL2/MCP1	Blood and CSF	Schröder et al., 2018 (#347)	Original research	10 PD, 13 HC	Increased	Significant elevation in CSF, exact value not reported	$p < 0.05$
CXCL12	Plasma	Li et al., 2022 (#348)	Original research	76 PD, 76 HC	Increased	36% elevation in people with PD	$p < 0.001$
CXCL12	Serum	Bagheri et al., 2018 (#351)	Original research	30 PD, 50 HC	Increased	133% elevation in people with PD (change inferred from figure)	$p < 0.0001$
sTNFR1	Serum and CSF	Qu et al., 2023 (#171)	Meta-analysis	9,032 PD, 12,628 HC (152 studies)	Increased	Hedges' g = 0.449	$p = 0.048$
NT-proBNP	Serum and CSF	Qu et al., 2023 (#171)	Meta-analysis	9,032 PD, 12,628 HC (152 studies)	Increased	Hedges' g = 0.533	$p < 0.001$
NT-proBNP	Blood	Choe et al., 2020 (#353)	Original research	285 PD, 570 HC	Increased	67% elevation in people with PD	$p < 0.001$
IFN-α2	Serum and CSF	Qu et al., 2023 (#171)	Meta-analysis	9,032 PD, 12,628 HC (152 studies)	Decreased	Hedges' g = -0.831	$P=0.008$
IL-4	Serum and CSF	Qu et al., 2023 (#171)	Meta-analysis	9,032 PD, 12,628 HC (152 studies)	Decreased	Hedges' g = -0.710	$P=0.026$
IL-4	Serum	Brodacki et al., 2008 (#185)	Original research	55 PD, 20 HC	Increased	7% elevation in people with PD (change inferred from figure)	$p < 0.001$
Nitric Oxide	Serum	Kouti et al., 2013 (#357)	Original research	58 PD, 15 HC	Increased	298% elevation in people with PD	$p < 0.0001$
Nitric Oxide	CSF	Qu et al., 2023 (#171)	Meta-analysis	9,032 PD, 12,628 HC (152 studies)	Increased	Hedges' g = 0.901	$p = 0.013$

Nitric Oxide	CSF	Santos-Lobato et al., 2022 (#358)	Original research	47 PD, 20 HC	Increased	F(2,47) = 3.25 for metabolite nitrite in CSF in people with PD	p = 0.04
						F(2,47) = 5.89 for metabolite nitrite in CSF in people with PD with dyskinesia	p = 0.005
Neuroendocrine Markers							
Cortisol	Serum	Breen et al., 2014 (#232)	Original research	30 PD, 15 HC	Increased	Increased 24hr-cortisol parameters in people with PD: acrophase (β = 0.502), amplitude (β = 0.485), and area under the curve (β = 0.615)	p < 0.002
						F(1,30) = 15.720	p < 0.001
Cortisol	Plasma	Hartmann et al., 1997 (#233)	Original research	12 PD, 10 HC	Increased	52% elevation in 24hr-pulsatile cortisol production rate in people with PD	p <0.05
						79% elevation in mass of cortisol secreted per burst in people with PD	p < 0.05
Cortisol	Saliva	Costa et al., 2019 (#234)	Original research	18 PD, 18 HC	Increased	56% elevation in 24-hr cortisol levels in people with PD	p = 0.03
Cortisol	Saliva	Skogar et al., 2011 (#235)	Original research	59 PD, 608 HC	Increased	28% elevation in daytime cortisol secretion in people with PD	p < 0.001
Cortisol	Saliva	Djamshidian et al., 2011 (#236)	Original research	13 PD, 14 HC	Increased	F(2,37) = 4.6	p = 0.016
Klotho	CSF	Sancesario et al., 2021 (#243)	Original research	26 PD, 9 HC	Increased in Early PD	F(1,33) = 4.3	p < 0.05
	Serum		Original research	22 PD, 9 HC	Decreased in Early PD	F(1,29) = 4.6	p < 0.05
Klotho	CSF	Zimmermann et al., 2021 (#241)	Original research	125 PD, 50 HC	Decreased	15% reduction in people with PD	p < 0.001
Klotho	Plasma	Kakar et al., 2021 (#242)	Original research	61 PD, 61 HC	Unchanged	None	n/a
Insulin	Plasma	Sanchez-Gomez et al., 2020 (#237)	Original research	73 PD, 38 HC	Decreased	20% reduction in fasting insulin in people with PD	p = 0.04

Insulin	Plasma	Hogg et al., 2018 (#266)	Original research	154 PD	Increased insulin resistance	58.4% of people with PD had undiagnosed, abnormal insulin resistance	n/a
Vitamin D	Serum	Evatt et al., 2011 (#238)	Original research	170 PD	Decreased	69.4% of people with PD had Vitamin D insufficiency or deficiency	n/a
Vitamin D	Plasma	Evatt et al., 2008 (#239)	Original research	100 PD, 99 HC	Decreased	14% reduction in 25(OH)D levels in people with PD; 53% prevalence of vitamin D insufficiency in people with PD	$p < 0.01$
Melatonin	Plasma	Videnovic et al., 2014 (#240)	Original research	20 PD, 15 HC	Decreased	4-fold decrease in amplitude of the circadian rhythm of melatonin and the 24-hour AUC	$p < 0.001$
Markers of PD Pathology							
α-synuclein	CSF	Grossauer et al., 2023 (#320)	Meta-analysis	1855 synucleinopathies, 1378 control	Positive SAA	0.88 sensitivity, 0.95 specificity	n/a
α-synuclein	CSF	Yoo et al., 2022 (#321)	Meta-analysis	2722 synucleinopathies, 2278 control	Positive SAA	0.88 sensitivity, 0.95 specificity	n/a
α-synuclein	CSF	Kang et al., 2019 (#312)	Original research	105 PD, 79 HC	Decreased	10% reduction in people with PD	$p = 0.009$
α-synuclein	CSF	Orru et al., 2020 (#314)	Original research	108 PD, 85 HC	Positive SAA	97% sensitivity, 87% specificity	n/a
α-synuclein	CSF	Majbour et al., 2021 (#317)	Original research	94 PD, 52 HC	Decreased	8% reduction in people with PD	$p < 0.05$
α-synuclein	CSF	Russo et al., 2021 (#318)	Original research	30 PD, 30 HC	Positive SAA	86-96% sensitivity, 93-100% specificity	n/a
α-synuclein	CSF	Poggiolini et a., 2022 (#319)	Original research	74 PD, 55 HC	Positive SAA	89% sensitivity, 96% specificity	n/a
α-synuclein	CSF	Kang et al., 2016 (#360)	Original research	412 PD, 189 HC	Decreased	13% reduction in people with PD	$p = 0.0002$
NFL	Serum	Mollenhauer et al., 2020 (#335)	Original research	397 PD, 187 HC	Increased	8% elevation in people with PD	$p = 0.03$

NFL	CSF	Mollenhauer et al., 2020 (#335)	Original research	98 PD, 61 HC	Increased	20% elevation in people with PD	p = 0.0001

PD, Parkinson's disease; HC, healthy control; CSF, cerebrospinal fluid; SNc, substantia nigra pars compacta; BDNF, brain-derived neurotrophic factor; GDNF, glial cell line-derived neurotrophic factor; IGF-1, insulin-like growth factor 1 (IGF-1); VEGF, vascular endothelial growth factor; NGF, nerve growth factor ; CDNF, cerebral dopamine neurotrophic factor; MANF, mesencephalic astrocyte-derived neurotrophic factor; GPLD1, glycosylphosphatidylinositol-specific phospholipase D1; TNF, tumor necrosis factor; CRP, C-reactive protein; sTNFR1, soluble TNF receptor-1; NT-proBNP, N-terminal pro-B-type natriuretic peptide; SMD, standardized mean difference; MD, mean difference; AUC, area under the curve; SAA, seed amplification assay

Supplemental Table 1B. Biomarkers Changes in Response to Aerobic Exercise

Biomarker	Sample Source	Study (reference number)	Type of Study	Study Population	Sample size (type of study)	Direction of Change with AE	Magnitude of Change	Associated P-value
Neurotrophic/Neuroprotective Markers								
BDNF	Blood	Szuhany et al. 2015 (#78)	Meta-analysis	Healthy adults and adults with chronic disease	703 (13 RCTs and single group intervention studies)	Increased	Hedges' g = 0.27	p = 0.005
BDNF	Blood	Gholami et al., 2025 (#79)	Meta-analysis	Healthy adults and adults with chronic disease	658 (14 RCTs)	Increased	SMD = 0.48	p = 0.037
BDNF	Serum	Rotondo et al. 2023 (#80)	Meta-analysis	Healthy adults and people with PD	180 (5 RCTs and single group intervention studies)	Increased	MD = 5.99 ng/mL	p = 0.04
BDNF	Blood	Paterno et al. 2024 (#81)	Meta-analysis	People with PD	185 (16 RCTS and NCTs)	Increased	SMD = 0.70	p = 0.04
BDNF	Serum	Kaagman et al. 2024 (#82)	Meta-analysis	People with PD	224 (5 RCTs)	Increased	SMD = 1.20	p = 0.0004
GDNF	Serum	Kong et al. 2024 (#97)	Original research	Healthy adults	160 (single RCT)	Increased	2% elevation	p = 0.027
IGF-1	Plasma	Baker et al. 2010 (#108)	Original research	Older men with MCI	15 (single RCT)	Increased	Not reported	p = 0.02
IGF-1	Plasma	Nasir et al., 2024 (#109)	Meta-analysis	Postmenopausal women	1170 (16 RCTs)	Increased	Effect size 3.132	p < 0.001
VEGF	Blood	Song et al. 2024 (#126)	Meta-analysis	Healthy adults	1097 (40 RCTs and single group intervention studies)	Increased	SMD = 0.18	p = 0.02

Irisin	Blood	Jandova et al. 2021 (#138)	Meta-analysis	Healthy adults and adults with chronic disease	1184 (59 RCTs and single group intervention studies)	Increased	SMD = 0.39	$p < 0.00001$
Irisin	Serum	Zhang et al. 2023 (#134)	Original research	People with PD	23 (single group intervention study)	Increased	12% elevation	$p = 0.041$
GPLD1	Plasma	Horowitz et al. 2020 (#142)	Original research	Healthy young adults	20 (observational study)	Increased in physically active adults	25% increased levels in active adults (inferred from figure)	$p < 0.05$
SIRT3	Skeletal muscle	Johnson et al. 2015 (#156)	Original research	Healthy older adults	23 (RCT)	Increased	6-fold elevation	$p = 0.012$
SIRT3	Skeletal muscle	Johnson et al. 2015 (#156)	Original research	Overweight adolescents	20 (RCT)	Increased	4-fold elevation	$p = 0.037$
SIRT3	Skeletal muscle	Vargas-Ortiz et al. 2015 (#157)	Original research	Overweight adolescents	14 (single group intervention study)	Increased	19% elevation	$p = 0.014$
Lactate	Blood	Cerexhe et al. 2022 (#167)	Original research	Adults with MS	59 (RCTs and single group intervention studies)	Decreased with submaximal AE and increased with maximal AE	SMD = -0.822 for lactate (sub-max); SMD = -0.54 for lactate (max)	$p < 0.001$
Lactate	Blood	Zhao et al. 2021 (#168)	Meta-analysis	Adults with Type II DM	43 (RCTs and single group intervention studies)	Decreased	SMD = -0.73	$p = 0.001$
Lactate	Blood	Di Martino et al. 2018 (#166)	Original research	People with PD	10 (single group intervention study)	Decreased	-34% lactate levels	$p < 0.05$
NGF	Blood	Lippi et al. 2020 (#346)	Systematic-review	Healthy adults and adults with chronic disease	292 (RCTs and single group intervention studies)	Inconclusive	n/a	n/a

Inflammatory Markers								
TNF	Blood	Khalafi et al., 2023 (#176)	Meta-analysis	Older adults	1898 (20 RCTs)	Decreased	SMD = -0.44	p = 0.009
TNF	Blood	Zheng et al., 2019 (#192)	Meta-analysis	Middle aged and older adults	1250 (11 RCTs and NCTs)	Decreased	SMD = 0.75	p = 0.0007
TNF	Blood	Papagianni et al., 2023 (#173)	Meta-analysis	Adults with Type II DM	1239 (26 RCTs)	Decreased	SMD = -2.70	p < 0.001
TNF	Blood	Hejazi et al., 2023 (#174)	Meta-analysis	Adults with Type II DM	1257 (25 RCTs)	Decreased	SMD = −1.20	p = 0.001
TNF	Blood	Del Rosso et al., 2023 (#209)	Meta-analysis	Overweight/obese adults	2422 (49 RCTs)	Decreased	Hedges' g = -0.491	p < 0.001
TNF	Blood	Zolandz et al., 2014 (#177)	Original research	People with PD	12 (single group intervention study)	Decreased	7% reduction	p = 0.03
TNF	Serum	Malczynska-Sims et al., 2022 (#178)	Original research	People with PD	28 (single NCT)	Decreased	22% reduction	p = 0.034
IL-6	Blood	Papagianni et al., 2023 (#173)	Meta-analysis	Adults with Type II DM	1239 (26 RCTs)	Decreased	WMD = -1.05	p < 0.001
IL-6	Blood	Hejazi et al., 2022 (#174)	Meta-analysis	Adults with Type II DM	1257 (25 RCTs)	Decreased	SMD = −1.08	p = 0.0001
IL-6	Blood	Zheng et al., 2019 (#192)	Meta-analysis	Middle aged and older adults	1250 (11 RCTs and NCTs)	Decreased	SMD = 0.75	p = 0.0007
IL-6	Blood	Del Rosso et al., 2023 (#209)	Meta-analysis	Overweight/obesity	3766 (25 RCTs)	Decreased	Hedges' g = -0.276	p=0.001
CRP	Blood	Khalafi et al., 2023 (#176)	Meta-analysis	Older adults	1898 (20 RCTs)	Decreased	SMD = -0.46	p = 0.04
CRP	Blood	Zheng et al., 2019 (#192)	Meta-analysis	Middle aged and older adults	1250 (11 RCTs and NCTs)	Decreased	SMD = 0.53	p = 0.0002
CRP	Blood	Papagianni et al., 2023 (#173)	Meta-analysis	Adults with Type II DM	1239 (26 RCTs)	Decreased	WMD = -0.91	p < 0.001
CRP	Blood	Hejazi et al., 2023 (#174)	Meta-analysis	Adults with Type II DM	1257 (25 RCTs)	Decreased	SMD = -0.67	p = 0.0006
CRP	Blood	Del Rosso et al., 2023 (#209)	Meta-analysis	Overweight/obesity	6476 (25 RCTs)	Decreased in obese adults	Hedges' g = -0.236	p < 0.001
IL-1β	Blood	Behboudi & Eizadi, 2017 (#210)	Meta-analysis	Overweight/obesity	6476 (25 RCTs)	Decreased with mixed AE and resistance exercise	Hedges' g = -0.968	p = 0.011

IL-1β	Serum	Del Rosso et al., 2023 (#209)	Original research	Sedentary obese women	30 (single RCT)	Decreased	23% reduction	p = 0.001
CX3CXL1 (Fractalkine)	Blood	Kumar et al., 2022 (#213)	Original research	Young & middle aged adults with mobility disability	38 (single group intervention study)	Decreased with mixed AE and resistance exercise	Not reported	p = 0.000054
CX3CXL1 (Fractalkine)	Blood	Njerve et al., 2016 (#214)	Original research	T2DM and stable coronary artery disease	114 (substudy of RCT)	Increased with mixed AE and resistance exercise only in people with advanced vascular disease	Not reported	p = 0.078
Clusterin	Plasma	De Miguel et al., 2021 (#216)	Original research	Veterans with MCI	20 (single group intervention study)	Increased	4% elevation	p<0.05
CCL2/MCP1	Blood	Trøseid et al., 2004 (#349)	Original research	Men with metabolic syndrome	22 (single RCT)	Decreased	Median difference = 48 pg/ml	p = 0.04
CCL2/MCP1	Blood	Clifford et al., 2023 (#350)	Original research	Cancer survivors	20 (single RCT)	Decreased with low intensity AE	MD = 13.2 pg/mL	p = 0.04
CXCL12	Blood	Clifford et al., 2023 (#350)	Original research	Cancer survivors	20 (single RCT)	Decreased with high intensity AE	MD = 150.3 pg/mL	p = 0.02
sTNFR1	Blood	Tsukui et al., 2000 (#352)	Original research	Healthy women	41 (single RCT)	Decreased	MD = 300 pg/mL	p < 0.01
NT-Pro-BNP	Blood	Bordbar et al., 2012 (#354)	Original research	Healthy adults	12 (single RCT)	Decreased	MD = 14.7 pg/mL	p = 0.013
NT-Pro-BNP	Serum	Malandish et al., 2022 (#355)	Systematic review and meta-analysis	Adults with heart failure	2563 (28 NCTs and RCTs)	Decreased	SMD = -0.336	p = 0.004
IL-4	Blood	Zheng et al., 2019 (#192)	Meta-analysis	Middle aged and older adults	1250 (11 RCTs and NCTs)	Unchanged	SMD = 0.00	p= 0.76

IL-4	Blood	Conroy et al., 2016 (#356)	Original research	Postmenopausal healthy women	720 (across 2 RCTs)	Unchanged	Study 1: 4.28% reduction Study 2: 8.24% reduction	Study 1: p= 0.54 Study 2: p= 0.32
Nitric Oxide	Serum	Arefirad et al., 2022 (#359)	Meta-analysis	Healthy adults and adults with chronic disease	136 (11 RCTs and NCTs)	Increased	Hedges' g = 1.82	p = 0.00
Neuroendocrine Markers								
Cortisol	Serum, saliva, urine	Beserra et al., 2018 (#244)	Meta-analysis	Major depressive disorder	209 (5 RCTs)	Decreased	SMD = -0.76	p < 0.001
Cortisol	Plasma	Baker et al., 2010 (#108)	Original research	Adults with MCI	33 (single RCT)	Decreased in women	$F_{1,25}$ = 6.00 (moderate effect on reduction of post-awakening cortisol)	p = 0.02
Cortisol	Saliva	Symth et al., 2019 (#261)	Original research	People with PD-MCI	8 (single group intervention study)	Decreased	19% reduction in post-awakening cortisol	p = 0.02
Klotho	Plasma, serum	Correa et al., 2022 (#252)	Meta-analysis	Healthy adults and adults with chronic diseases	92 (5 RCTs)	Increased	Hedges' g = 0.92	p < 0.0001
Insulin	Plasma	Sampath Kumar et al., 2019 (#248)	Meta-analysis	Adults with Type II DM	175 (4 RCTs)	Decreased insulin resistance	Chi2 = 26.50	p < 0.00001
Insulin	Plasma	Mann et al., 2014 (#275)	Meta-analysis	Adults with Type II DM or metabolic syndromes/abnormalities	845 (16 RCTs and single group intervention studies)	Increased insulin sensitivity	Effect size = 1.07	not reported

Vitamin D [25(OH)D]	Serum	Zhang & Cao, 2022 (#249)	Systematic review	Healthy adults, adults with conditions such as osteoporosis, COPD, overweight/obesity	910 (12 RCTs and single group intervention studies)	Increased in people with vitamin D deficiency	Significant increase in most studies	not reported
Melatonin	Serum	Al-Rawaf et al., 2023 (#246)	Original research	Healthy adults	80 (single group intervention study)	Increased	3-fold increase	p = 0.001
Markers of PD Pathology								
NFL	Serum	Ercan et al., 2021 (#339)	Original research	Relapsing remitting multiple sclerosis	38 (single RCT)	Decreased	32% reduction	p = 0.02
NFL	Serum	Frederiksen et al., 2023 (#340)	Original research	Alzheimer's disease	136 (single RCT)	Unchanged	n/a	n/a

PD, Parkinson's disease; AE, aerobic exercise; MCI, mild cognitive impairment, DM, diabetes mellitus; BDNF, brain-derived neurotrophic factor; GDNF, glial cell line-derived neurotrophic factor; IGF-1, insulin-like growth factor 1 (IGF-1); VEGF, vascular endothelial growth factor; NGF, nerve growth factor ; CDNF, cerebral dopamine neurotrophic factor; MANF, mesencephalic astrocyte-derived neurotrophic factor; GPLD1, glycosylphosphatidylinositol-specific phospholipase D1; TNF, tumor necrosis factor; CRP, C-reactive protein; sTNFR1, soluble TNF receptor-1; NT-proBNP, N-terminal pro-B-type natriuretic peptide; SMD, standardized mean difference; MD, mean difference; RCT, randomized controlled trial; NCT, non-randomized controlled trial
